# 23^rd^ International Colloquium on Animal Cytogenetics and Genomics (23 ICACG) June 9–12, 2018, Saint-Petersburg, Russia

**DOI:** 10.3897/CompCytogen.v12i3.27748

**Published:** 2018-08-16

**Authors:** Svetlana Galkina, Maria Vishnevskaya

**Affiliations:** 1 St Petersburg State University, St. Petersburg, Russia St. Petersburg State University St. Petersburg Russia

## Abstract

In memory of Ingemar Gustavsson

23^rd^ International Colloquium on Animal Cytogenetics and Genomics (23 ICACG) took place in June 9–12, 2018 in Saint-Petersburg, Russia. Organized biennially, the Colloquium runs from 1970. From its very start this meeting is associated with the name of Ingemar Gustavsson to whom we dedicated the Colloquium 2018. The long and productive career of Ingemar Gustavsson had focused on chromosomes and their fundamental role in animal physiology, fertility, health and production in the context of agriculture and veterinary medicine. His meticulous analysis of breeding data performed back in 1964–69 resulted in the unequivocal identification of an association between heterozygosity for the 1/29 translocation in Swedish cattle and reduction in the fertility of the breed. Eventually, the argument in favor of selective elimination of bulls carrying the translocation from the breeding programs prevailed and the field of modern veterinary cytogenetics was established.

Participants from fourteen different countries attended the 23 ICACG in Russia, the country having long lasting traditions in cytogenetics and the Scientific schools of N.K. Koltzov, S.S. Chetverikov and A.S. Serebrovsky, geneticists who made important conceptual contributions to studies of chromosomes and genes, population genetics and evolutionary theory as early as in the beginning of the XX-th century.

All the abstracts received were subdivided between plenary and seven scientific sessions covering the issues in evolutionary and comparative cytogenetics, cytogenetics and genomes of domestic animals, meiosis studies, particular chromosome analyses, clinical cytogenetics, karyotypes and genomes of vertebrate and invertebrate animals, chromatin studies. In the abstract text below each presentation is marked with a capital letter: „**L**” stands for lectures, „**O**” for oral presentations and „**P**” for poster presentations.

We gratefully acknowledge the support from the Saint-Petersburg Association of Scientists and Scholars (SPbSU), Veterinary Genetics Center ZOOGEN, Russian Foundation for Basic Research (RFBR), VEUK, Helicon, Axioma BIO, BioVitrum, Sartorius, DIA-M companies.

The current collected abstracts comprise written contributions of the presentations during the 23 ICACG and were edited by Svetlana Galkina and Maria Vishnevskaya.

The next Colloquium – 24 ICACG – will be held at the University of Kent in Canterbury (UK) in 2020.

Please, cite abstracts as follows:

Gall JG (2018) Giant chromosomes and deep sequences: what the amphibian egg tells us about transcription. In: Galkina SA, Vishnevskaya MS, Mikhailova EI (Eds) 23^rd^ Inernational Colloquium on Animal Cytogenetics and Genomics (23^rd^ICACG), June 9–12, 2018, St Petersburg, Russia. Comparative Cytogenetics 12(3): p–p. https://doi.org/10.3897/CompCytogen.v12i3.27748

## Plenary session

### L1

#### Giant chromosomes and deep sequences: what the amphibian egg tells us about transcription

Joseph G. Gall

*Department of Embryology, Carnegie Institution for Science, 3520 San Martin Drive, Baltimore MD 21218 USA* (*gall@carnegiescience.edu*)

Lampbrush chromosomes (LBCs) occur in oocytes during prophase of the first meiotic division. They are characterized by extremely large transcription units (TUs), which extend as loops up to 100 mkm or more in length from an axis of condensed chromatin. The length of the TUs reflects the extraordinary close packing of RNA polymerase II molecules along the extended DNA, indicative of an extremely high rate of transcription. LBCs are of very wide occurrence phylogenetically, but are limited to large oocytes in which the oocyte nucleus is the sole source of maternal mRNA. Simple calculations show that a giant cell must either (1) become polyploid or (2) transcribe at an extraordinary rate to supply the needed mRNA in a reasonable time (days or weeks rather than years). Giant somatic cells are almost universally polyploid. Giant oocytes, on the other hand, cannot become polyploid, since they must undergo meiosis. It is probable, therefore, that LBCs represent the means by which a 4C oocyte nucleus can transcribe sufficient mRNA (in a reasonable time) to meet the needs of a giant oocyte.

Within this broad theoretical framework, there remain numerous unanswered questions about the LBC loops. What sequences are transcribed on the loops, as opposed to sequences that accumulate as stable mRNA in the cytoplasm? Where precisely are the promoter regions of the genes? Is there read-through transcription, especially on loops that contain two or more TUs? Although splicing is clearly co-transcriptional, are the introns shed from the loops before the end of the TU? What holds the bases of the loops together? What is the relationship, if any, of LBC loops to the TADs defined by Hi-C experiments?

In this lecture I will outline recent studies on LBCs and discuss new experimental approaches that can be applied to answer some of these outstanding questions.

## L2

### A moving landscape for comparative genomics in mammals

Stephen J. Obrien, Gaik S. Tamazian, Aleksey S. Komissarov, Pavel V. Dobrynin, Ksenia V. Krasheninnikova, Sergey F. Kliver, Nikolay A. Cherkasov, Klaus P. Koepfli

*Theodosius Dobzhansky Center for Genome Bioinformatics, St Petersburg State University, 41 Sredniy Prospekt, St. Petersburg, Russia, 199004*

Corresponding author: *Stephen J. Obrien* (*lgdchief@gmail.com*)

Today we count some 62,000 species of vertebrates (half are fishes) including some 550 species of mammals on earth. The genome sequencing of non-laboratory species in recent years is expanding our breadth and understanding of genetic bases of adaptation and evolution in varied and amazing ways.

Recent completion and inspection of whole genome sequence and assembly for over 200 species of mammals, from platypus to panda to human, offer the prospect of a better view of the patterns of changes within genome organization across the mammalian radiations. In 2009 my colleagues and I have created Genome-10K, an international consortium of scientist who have set a goal of gathering, sequencing, assembling, and annotating to high quality some 10,000 vertebrate genomes with 2^nd^ and 3^rd^ generation sequencing technology within the coming five years. These activities and advances provide an enormous Bioinformatics challenge whose solution will provide future zoologists of every persuasion a genome sequence resource for their favorite study animal. The applications and potential for the genome sequence in several research questions will be discussed.

**Acknowledgements**

The study has been supported by the grant from Russian Science Foundation (17-14-01138).

## L3

### Chromosome abnormalities in domestic bovids: a review

Leopoldo Iannuzzi

*Institute for Animal Production System in Mediterranean Environment* (*ISPAAM*), *National Research Council* (*CNR*), *via Argine 1085, Naples, 80147, Italy* (*leopiannuzzi949@gmail.com*)

After the discovery of rob(1;29) in the Swedish red cattle breed (Gustavsson and Rockborn 1964) and the demonstration of its deleterious effect on the fertility (Gustavsson 1969, 1971; Dyrendahl and Gustavsson 1979), the cytogenetics applied on domestic animals has been widely expanded in many laboratories to find relationships between chromosome abnormalities and phenotypic effects, especially on fertility. However, in the same years of this discovery, various groups of cytogeneticists published several reports on chromosome abnormalities, primarily involving sex chromosomes, underlining the importance of these types of abnormalities, often responsible for sterility, especially in the females. While numerical autosome abnormalities have been rarely reported, being animal phenotypes abnormal and easily eliminated directly by breeders, numerical sex chromosome abnormalities, as well as structural (and balanced) chromosome anomalies have been more frequently found in domestic bovids because they are often phenotypically invisible for breeders. For this reason, these chromosome abnormalities, without a cytogenetic control, escape selection leading to subsequent deleterious effects on the fertility, especially in females carrying sex chromosome abnormalities. In addition, chromosome abnormalities can be easily spread in the progeny, especially when an artificial insemination is utilized. Certainly, an advent of chromosome banding techniques and application of the FISH-mapping technique with the use of specific molecular markers (generally BAC-clones) and/or chromosome painting probes (Zoo-FISH), proved to be a powerful tool for cytogeneticists in their daily work of identification of the specific chromosomes affected by the abnormalities, especially when the banding pattern resolution is poor (as in many published papers, especially in the past). However, very few groups are actually involved in clinical cytogenetic analysis applied to domestic animal breeding. This problem needs to be addressed because clinical cytogenetics still remains one of the most important aspect of our work, especially for breeders and, in a longer run, for a genetic improvement of the livestock in general. In this brief review I will present a list of the most important chromosome abnormalities found in domestic bovids (mainly in cattle, sheep and river buffalo), and will suggest the strategies for the better detection of these rearrangements in animal populations by employment of efficient and simple banding techniques allowing to speed up the analyses and obtaining desirable results.

**Acknowledgements**

The study has been supported by the project PON1_486 GENOBU

## L4

### Viewing nuclear architecture through the eyes of nocturnal mammals

Irina Solovei

*Department of Biology II, Biozentrum, Ludwig-Maximilians University Munich, Grosshadernerstrasse 2, 82152 Planegg-Martinsried, Germany* (*irina.solovei@lrz.uni-muenchen.de*)

The mammalian nucleus displays spatial segregation of active euchromatic from inactive heterochromatic genomic regions. In conventional nuclei, euchromatin is localized in the nuclear interior, whereas heterochromatin lies at the nuclear periphery. In contrast, rod photoreceptors of nocturnal mammals have inverted nuclei, with a dense heterochromatic core and a thin euchromatic outer shell. This inverted architecture converts rod nuclei into microlenses reducing light scattering in thick nocturnal retinas and thus facilitating vision at scotopic conditions.

The unusual nuclear organization of nocturnal rods with a regular concentric arrangement of different chromatin classes offers a unique model to study various aspects of nuclear architecture. Within the last decade, studies of inverted nuclei helped us to understand several fundamental principles of nuclear architecture:

(i) We have demonstrated that although inverted nuclei are fully functional, inversion is not a favorable chromatin arrangement in mammalian cells (Solovei et al. 2009)

(ii) We have identified two major mechanisms of heterochromatin peripheral tethering that are responsible for establishing the conventional nuclear architecture (Solovei et al. 2013)

(iii) We have shown that the building of a functional nucleus is largely a self-organizing process based on mutual recognition of chromosome segments marked by certain repeats (van de Werken et al. 2017)

(iv) Finally, we have recently demonstrated that interactions among heterochromatic regions are central to phase separation of the active and inactive genome in inverted and conventional nuclei (Falk et al. 2018).

## Evolutionary and comparative cytogenetics

### L5

#### Chromosome-specific sequencing in comparative genomics

Malcolm A. Ferguson Smith

*Cambridge University Department of Veterinary Medicine, Madingley Road, Cambridge CB3 0ES, UK* (*maf12@cam.ac.uk*)

Twenty-five years ago the introduction of chromosome painting revealed chromosome homology between species and provided a powerful method to determine phylogenetic relationships among vertebrates. It showed that genome conservation occurs in large homologous syntenic blocks (HSBs) and that the assembly of HSBs in karyotypes differs between species. Chromosome sorting and next generation sequencing now provides a more precise method for comparative genomics. Sequence reads from chromosome-specific DNA can be aligned to total genome sequence of other species available from the genome database. This reveals chromosome homology at the nucleotide level and defines evolutionary recombination breakpoints. The procedure has helped in the determination of chromosome homology between human and camelids where conventional painting has proved challenging.

## L6

### Molecular cytogenetics in Zoo-FISH-studies – still urgently needed

Thomas Liehr

*Jena University Hospital, Friedrich Schiller University, Institute of Human Genetics, Am Klinikum 1, D-07747 Jena, Germany* (*Thomas.Liehr@med.uni-jena.de*)

Evolution can be observed, e.g. in different species of the same order or family, on different levels of resolution. Here I want to highlight the importance of all available approaches; chromosomal studies are the bases for all higher-resolution based approaches. Knowledge about chromosome number of a species, basic structure of chromosomes and (molecular) cytogenetically detectable gross chromosomal rearrangements compared to an outgroup is necessary first. Without that, wrong conclusions will be drawn from high resolution molecular data. This is among others due to the fact that high throughput sequencing still has problems in correct alignment of repetitive sequences.

## L7

### Evolutionary sex chromosome translocations in amniotes

Vladimir A. Trifonov^1,2^, Artem P. Lisachov^3^, Ilya G. Kichigin^1^, Alexey I. Makunin^1^, Jorge C. Pereira^4^, Anna S. Druzhkova^1^, Malcolm A. Ferguson-Smith^4^, Massimo Giovannotti^5^

**1** *Institute of Molecular and Cellular Biology, Russian Academy of Sciences, Siberian Branch, Novosibirsk 630090, Russia* **2** *Novosibirsk State University, Novosibirsk 630090, Russia* **3** *Institute of Cytology and Genetics, Russian Academy of Sciences, Siberian Branch, Novosibirsk 630090, Russia* **4** *Cambridge Resource Centre for Comparative Genomics, Department of Veterinary Medicine, University of Cambridge, Madingley Road, Cambridge CB3 0ES, UK* **5** *Dipartimento di Scienze della Vita e dell’Ambiente, Università Politecnica delle Marche, via Brecce Bianche, 60131 Ancona, Italy*

Corresponding author: *Vladimir Trifonov* (*vlad@mcb.nsc.ru*)

High diversity of sex determination mechanisms in reptiles versus conserved sex chromosomes in therian mammals and birds is usually explained by a rapid turnover of sex determining pathways in cold-blooded vertebrates. However, pleurodont lizards represent an interesting exception, where an unusually conserved system of sex chromosomes has originated over 160 million years ago. Opposite to mammals and birds, where sex chromosomes only rarely translocate on autosomes, the ancestral sex chromosomes of dactyloid lizards have undergone fusions with one or several microchromosomes in different lineages, thus representing an interesting model to study the evolution of autosomal blocks after their transposition to sex chromosomes. Here, using low pass sequencing chromosome specific probes, we study the genetic content and evolution of sex chromosomal systems in several lineages of anoles (Dactyloidae). We demonstrate that autosomal elements undergo partial degeneration and accumulate specific repeated elements after their fusion with sex chromosomes. We postulate that the translocation of autosomal blocks onto sex chromosomes may have facilitated rapid degeneration of the pseudoautosomal region on the ancestral Y. The enrichment in repetitive DNA in chromosome specific DNA pools may serve as an indication of sex chromosomes in species with cytologically indistinguishable micro-sex chromosomes.

**Acknowledgements**

The work was financially supported by RSF grant No. 16-14-10009.

## O1

### Comparative genomics through the development of universal cross-species BAC sets

Rebecca E. O’Connor^1^, Marta Farré^2^, Lucas Kiazim^1^, Rebecca Jennings^1^, Joana Damas^2^, Denis M. Larkin^2^, Darren K. Griffin^1^

**1** *University of Kent, Canterbury, CT2 7NJ, UK* **2** *Royal Veterinary College, University of London, London NW1 0TU, UK*

Corresponding author: *Rebecca E. O’Connor* (*ro215@kent.ac.uk*)

Comparative genomics using targeted BAC based FISH (fluorescence *in situ* hybridization) probes, is generally restricted to closely related species due to extensive sequence divergence between distantly related species. The ability to identify precise regions of homology between species (for genome mapping, phylogenomics and genome organization studies) and the ability to anchor genomic sequence data to chromosomes (for chromosome assembly) is therefore restricted.

To overcome these difficulties, we developed a set of universal avian BAC probes, selected through the identification of evolutionary conserved regions. This BAC set was then used to upgrade the genomes of 5 avian species to a chromosome-level. Successful hybridisation of these probes to a further ~30 avian species revealed genome-wide patterns of chromosome stability and rearrangement between species. In addition, the probes successfully hybridized on non-avian reptile species (turtles and anole lizard) revealing a level of genome conservation extending far beyond birds.

Further, we applied the approach developed for avian probes to the selection of BACs from the cattle and human genome with the aim of generating a universal mammalian BAC set. Selection criteria were validated by testing probes on species at key nodes of the phylogenetic tree. Hybridisations were achieved on species as diverse as horse *Equusferus* Boddaert, 1785, dolphin *Tursiopsaduncus* Ehrenberg, 1833, bat *Lophostomasilvicolum* D’Orbigny, 1836 and lemur *Eulemurmacaco* Linnaeus, 1766.

These preliminary results illustrate that our combined FISH-bioinformatics approach is also applicable to mammals. Development of a universal BAC set therefore permits cross-species sequence anchoring and comparative genomic research at a higher resolution than previously possible, providing new insight into the nature of genomic evolution and genomic stability.

## O2

### Pinniped karyotype evolution substantiated by comparative chromosome painting of 10 pinniped species (Pinnipedia, Carnivora)

Violetta R. Beklemisheva^1^, Polina L. Perelman^1,2^, Natalia A. Lemskaya^1^, Anastasia I. Kulemzina^1^, Anastasia A. Proskuryakova^1,2^, Vladimir N. Burkanov^3,4^, Steven J. O’Brien^5,6^, Alexander S. Graphodatsky^1,2^

**1** *Department of Comparative Genomics, Institute of Molecular and Cellular Biology, SB RAS, Acad. Lavrentiev Ave. 8/2, Novosibirsk, 630090, Russia* **2** *Novosibirsk State University, 1, Pirogova str., Novosibirsk, 630090, Russia* **3** *Department of Higher Vertebrates Ecology, Kamchatka Branch of Pacific Geographical Institute FEB RAS, 6, Partizanskaja str., Petropavlovsk-Kamchatski, 683000, Russia* **4** *National Marine Mammal Laboratory, AFSC, NMFS, 7600 Sand Point Way N.E., Building 4 Seattle, Washington, 98115, USA* **5** *Theodosius Dobzhansky Center for Genome Bioinformatics, 41A, Sredniy Av., St. Petersburg State University, St. Petersburg, 199034, Russia* **6** *Oceanographic Center, Nova Southeastern University, 3301 College Avenue, Fort Lauderdale, Florida 33314-7796, USA*

Corresponding author: *Violetta R. Beklemisheva* (*bekl@mcb.nsc.ru*)

Numerous Carnivora karyotype evolution investigations have been performed by classical and molecular cytogenetics and were supplemented by reconstructions of the Ancestral Carnivora Karyotype (ACK). However, the group of Pinnipedia was not studied in detail. Here we reconstruct pinniped karyotype evolution and refine ACK using published and our new painting data for 10 pinniped species. The combination of human (HSA) and domestic dog (CFA) whole-chromosome painting probes was used for the construction of the comparative chromosome maps for species from all three pinniped families: Odobenidae – *Odobenusrosmarus* Linnaeus, 1758, Phocidae – *Phocavitulina* Linnaeus, 1758, *Pusasibirica* Gmelin, 1788, *Erignathusbarbatus* Erxleben, 1777, *Phocalargha* Pallas, 1811, *Phocahispida* Schreber, 1775 and Otariidae – *Eumetopiasjubatus* Schreber, 1775, *Callorhinusursinus* Linnaeus, 1758, *Phocarctoshookeri* Gray, 1844, *Arctocephalusforsteri* Lesson, 1828. HSA and CFA autosome painting probes have delineated 32 and 68 conservative autosome segments in the studied genomes. The comparative painting in Pinnipedia supports monophyletic origin of pinnipeds, shows that pinniped karyotype evolution was characterized by slow rate of genome rearrangements (less then one rearrangement per 10 million years), provides strong support for refined structure of ACK with 2n = 38 and specifies plausible order of dog chromosome synthenic segments on ancestral Carnivora chromosomes. The heterochromatin, telomere and ribosomal DNA distribution was studied in all 10 species.

**Acknowledgements**

The work was financially supported by RFBR №17-00-00146, RSF №16-14-10009.

## O3

### X chromosome evolution in Cetartiodactyla

Anastasia A. Proskuryakova^1,2^, Anastasia I. Kulemzina^1^, Polina L. Perelman^1,2^, Alexey I. Makunin^1^, Natalya A. Lemskaya^1^, Violetta R. Beklemisheva^1^, Denis M. Larkin^3^, Marta Farre^3^ Anna V. Kukekova^4^, Oliver A. Ryder^5^, Stephen J. O’Brien^6^, Alexander S. Graphodatsky^1,2^

**1** *Institute of Molecular and Cellular Biology, SB RAS, Lavrentjeva ave 8/2, Novosibirsk, 630090, Russian Federation* **2** *Novosibirsk State University, Pirogova str. 1, Novosibirsk, 630090, Russian Federation* **3** *The Royal Veterinary College, Royal College Street, University of London, London NW1 0TU, UK* **4** *The University of Illinois at Urbana-Champaign, W. Gregory Drive Urbana 1207, Illinois, USA* **5** *Zoological Society of San Diego, Conservation and Research for Endangered Species, San Pasqual Valley Road 15600, Escondido, CA 92027, USA* **6** *Theodosius Dobzhansky Center for Genome Bioinformatics, Saint-Petersburg State University, Sredniy Av. 41A, Saint-Petersburg, 199034, Russia*

Corresponding author: *Anastasia A. Proskuryakova* (*andrena@mcb.nsc.ru*)

The mammalian X chromosome is characterized by high level of conservation. On the contrary the Cetartiodactyl X chromosome displays variation in morphology and G-banding pattern. It is hypothesized that X chromosome has undergone multiple rearrangements during Cetartiodactyla speciation. To investigate the evolution of this sex chromosome we have selected 26 BAC clones from cattle CHORI-240 library evenly distributed along the cattle X chromosome. High-resolution maps were obtained by fluorescence *in situ* hybridisation in a representative range of cetartiodactyl species from different families: pig (Suidae), gray whale (Eschrichtiidae), pilot whale (Delphinidae), hippopotamus (Hippopotamidae), Java mouse deer (Tragulidae), pronghorn (Antilocapridae), Siberian musk deer (Moschidae), giraffe (Giraffidae). To trace the X chromosome evolution during fast radiation in speciose families, we mapped more than one species in Cervidae (moose, Siberian roe deer, fallow deer and Pere David’s deer) and Bovidae (musk ox, goat, sheep, sable antelope, nilgau, gaur, saola, and cattle). We have identified three major conserved synteny blocks and based on this data reconstructed the structure of putative ancestral cetartiodactyl X chromosome. We demonstrate that intrachromosomal rearrangements such as inversions and centromere reposition are main drivers of cetartiodactyl’s chromosome X evolution.

**Acknowledgements**

The work was supported by the Russian Science Foundation (RSF #16-14-10009).

## Cytogenetics and genomics of domestic animals

### L8

#### Copy number variations in cattle and pigs: aging and reproduction

W. Allan King^1,2^, Olutobi Oluwole^1^, Tamas Revay^1^

**1** *Department of Biomedical Sciences, University of Guelph, 50 Stone rd. Guelph, ON, Canada N1G2W1* **2** *Karyotekk Inc, University of Guelph, 50 Stone rd. Guelph, ON, Canada N1G2W1*

Corresponding author: *W. Allan King* (*waking@uoguelph.ca*)

Variability in genomes (including single nucleotide polymorphisms (SNP), copy number variations (CNV) and chromosomal rearrangements (CA)) is responsible for a significant proportion of the diverse phenotypes associated with many important traits including fertility and ageing. We have investigated copy number variation in Canadian Holstein bulls and Yorkshire, Landrace and Duroc boars. In bulls we studied *de novo* CNVs from two perspectives: somatic variability and ageing. In boars we compared CNVs between those with very high or very low DBE (direct boar effect) on litter size.

Somatic tissues (blood, lung, heart, muscle, testis, brain) from four Holstein bulls were sampled, arrayed on the Bovine SNP50k chip and genome-wide CNVs analyzed from the probe intensity data. The results showed extensive copy number divergence among tissues of the same animal.

We have detected a median 31 CNVs per animal which were classified as either germ line origin (median 14), as they were constantly present in all investigated tissues of the animal or *de novo* somatic (median 18), those specific to one tissue. Thus, 57% of the total number of detected CNVs was the result of *de novo* somatic events. Additionally, CNVs from blood of 8 bulls at 3 different time points (14±3, 32±3, and 44±3 month of age) were examined, in just under 3 years *de novo* CNVs were generated in almost equal number to the number present at the start and throughout the study (107 vs. 111 or 49% vs. 51%, respectively), which were regarded as constant CNVs. Also, twice as many *de novo* CNVs emerged during the second half of the sampling schedule as in the first part (a total of 71 vs. 36, respectively).

Two groups of boars were selected from the upper and lower 10% of DBE value distribution of more than 38,000 animals and genotyped with the Porcine SNP60K chip. The CNV analysis identified 35 CNVs covering 36.5 Mb or ~1.3% of the porcine genome that were specific to the high fertility group (14) or to the low fertility group (19). These CNVs overlapped with 137 QTLs of reproductive traits and also with 50 genes significantly enriched in members of the innate immune system, various receptor signaling pathways and fatty acid oxidation. These genomic regions and physiological functions could be connected to fertility, thus might represent putative markers.

The results suggest that genome of cattle and pigs is dynamic and in constant change. Changes such as copy number variation are associated with fertility and aging.

**Acknowledgements**

Research support was obtained from NSERC, Agriculture and Agri-Food Canada, Ontario Ministry of Agriculture and Rural Affairs, and the Canada Research Chairs program.

## L9

### Whole genome genotyping and sequencing reveal history and signatures of adaptations in genomes of Russian native cattle breeds

Denis M. Larkin^1,2^

**1***The Federal Research Center Institute of Cytology and Genetics, The Siberian Branch of the Russian Academy of Sciences, Prospect Lavrientieva 10, Novosibirsk, 630090, Russia***2***Royal Veterinary College, University of London, 4 Royal College Street, NW1 0TU, London, UK* (*dmlarkin@gmail.com*)

In Russia ~20 native cattle breeds are currently being recognized. We focused on revealing history of their formation and signatures of genomic selection. We genotyped 274 animals from 18 Russian breeds on the GGP HD150K and 50K Bovine SNP arrays and combined the data with additional 135 cattle breeds. The data were clustered to reveal relationship between the breeds. Signatures of selection were detected using the hapFLK software and the de-correlated composite of multiple signals framework combining the FST, H1, H12, Tajima’s D, nucleotide diversity statistics. To reveal specific nucleotide variants that could be related to economically important traits and adaptation to extreme environment, the two most distant breeds (Yakut, Kholmogory) were sequenced (40 animals) and signatures of selection/adaptation were detected. Four major clusters involving Russian breeds were identified. Yakut cattle were the most divergent amongst all taurine breeds in our dataset. The strongest signals of selection in Russian cattle were detected near the genes: LCORL/NCAPG, HMGA2, IMPAD1 (growth), KIT (coat colour), PLAG1 (reproduction). We further detected signatures of selection related to domestication (KITLG, EDN3, COPA), feed intake (XKR4, TMEM68), milk production (DGAT1, GHR, ABCG2, GLI2, LAP3, TRPV5, FKBP2), and reproduction (CSF2, BCL2, ANXA10, NPBWR1). Strong candidate genes for adaptation to cold climate and local environment were found under selection in the Yakut cattle: RETREG1, RPL7, TNKS, Kholmogory: AQP5, and multiple Russian breeds: ARRDC3, RAD50, SYK. Multiple regions under selection identified in the set of Russian native cattle breeds form a basis for future genomics-based selection and targeted breeding of Russian cattle.

**Acknowledgements**

This study was supported by the grant from the Russian Science Foundation (project no. 16–14-00090).

## O4

### An innovative method of gene co expression network inference reveals significant biological processes involved in fetal porcine muscle development

Maria Marti-Marimon^1^, Nathalie Villa-Vialaneix^2^, Valentin Voillet^1^, Martine Yerle-Bouissou^1^, Laurence Liaubet^1^, Yvette Lahbib-Mansais^1^

**1** *GenPhySE and* **2** *MIAT, University of Toulouse, INRA Occitanie-Toulouse, ENVT, 24 chemin de Borde-rouge 31326 Castanet Tolosan, France*

Corresponding author: *Yvette Lahbib-Mansais* (*yvette.lahbib-mansais@inra.fr*)

The integration of genetic information in the cellular and nuclear environments is crucial for deciphering how the genome functions in physiological conditions. By combining 3D nuclear mapping, high-flow transcriptomic data analyses, and statistical methods for the development of co-regulated gene networks, it becomes possible to develop an integrated approach to depict the regulation of gene expression. For this purpose, we focused on the mechanisms involved in the transcriptional regulation of genes expressed in muscle during late fetal development in pig (90 and 110 days), a critical period for survival. We published a muscle transcriptomic analysis performed during this perinatal period (Voillet et al. 2014). Data from this previous study obtained from two extreme genetic lines in terms of mortality at birth (Large White and Meishan), were used to construct networks of differentially co-expressed genes. As co-expressed genes are not necessary related to a common biological process, we used information of gene co-localizations (3D DNA FISH) to reinforce observed links in the co-expressed gene network. The innovative network inference method developed, sequentially incorporates biological knowledge on gene spatial co-localization to construct robust networks gathering co-regulated genes. Clustering of nodes (genes) becomes more and more biologically consistent in each iteration. Interestingly, by means of the final network, we particularly uncovered unexpected gene associations in the nuclear space between IGF2 and MYH3 suggesting that they could be subject to similar transcriptional regulation.

## O5

### Assessment of DNA damage in a rare case of ewe-buck hybrid using the comet assay

Alfredo Pauciullo^1^, Georg Erhardt^2^, Liliana Di Stasio^1^, Isabel Wiedemann^3^, Christoph Knorr^3^

**1***University of Turin, Department of Agricultural, Forest and Food Sciences, Largo Paolo Braccini 2, Grugliasco* (*To*), *Italy***2***Justus-Liebig-University of Giessen, Institute of Animal Breeding and Genetics, Ludwigstrasse 21b, Giessen, Germany***3***Georg-August-University, Department for Animal Science, Biotechnology and Reproduction of Livestock, Burckhardtweg 2, Göttingen, Germany*

Corresponding author: *Alfredo Pauciullo* (*alfredo.pauciullo@unito.it*)

Over the past decade, the Comet assay has been used in humans to study processes ranging from DNA repair to genotoxicity, mainly to assess the adverse effects of toxins on DNA. In contrast to humans, there are only few reports on comet assay in livestock. In the present study, we assessed the Comet assay to investigate the genome stability and detect primary DNA damages in a very rare case of an ewe-buck hybrid (2n=57, XX). Three contemporary sheep (breed Leineschafe) (2n=54, XX) and three goats (Weiße Deutsche Edelziege) (2n=60, XX) belonging to the same flock were used as controls.

Blood samples were collected in K_2_EDTAtubes. Comet assays (under alkaline conditions) were performed as described by Singh et al. (1988). Shortly, 1x10^5^ lymphocyte cells/mL were combined with molten LMAgarose (at 37 °C) at 1:10 (v/v) ratio. Alkaline electrophoresis was performed at 4 °C for 30 min at 300mA, followed by staining with SYBR^®^ Green I. The slides were observed at 200x magnification with a Nikon Eclipse 80i fluorescence microscope equipped with FITC specific filter and provided with a LAS image-analysis system. Two slides per sample were analyzed and a total of 50 individual cells per animal were randomly chosen and screened per subject (25 cells from each slide). OpenComet software was used for scoring the comet characteristics, including tail length, tail moment, head area.

Departures from symmetry (P<0.05) were observed for each data distribution. Therefore, non-parametric tests (Kruskal-Wallis and Dunn) with Bonferroni correction were employed for the statistical analysis. Differences between means were considered statistically significant for P<0.05.

The hybrid animal showed significantly greater mean DNA tail length (16.42 ± 3.73 µm) than sheep (P<0.0001) and goat (P<0.025). In addition, the hybrid’s tail moment (product of the tail length and the fraction of total DNA in the tail) was higher (4.76±2.68) compared to the sheep (P<0.0001) and goat (P<0.010). By contrast, the mean value of the head area (7095 ± 248 µm^2^), was intermediate between sheep (P<0.0001) and goat (P<0.0001) was detected. The data reveal a higher DNA damage of the hybrid animal that is most likely due to the nature of its genetic diversity, especially as we consider the absence of the main environmental effects among the investigated animals (similar age, flock, feeding system). Reference data are not available to compare our analysis with the literature. Therefore, further investigations (more data from the comet assay and additional tests like SCE, CA, etc.) are necessary for deeper understanding the stability of the hybrid genome.

## O6

### Routine diagnostics in companion animals by digital PCR

Darja Krylova

*Independent Veterinary Laboratory “Poisk”, Repisheva street 13, Saint-Petersburg, Russia* (*amenshenin1975@mail.ru*)

Some of the small domestic animal diseases were diagnosed until recently only by qualitative or semi-quantitative PCR methods. Furthermore, effectiveness of a given kind of treatment was evaluated by clinical scores, and sometimes it was affected by comorbidities. Development of the digital PCR method made it possible to precisely control the influence of certain treatment mode on disease state at the molecular level. The benefits of using digital PCR are obvious as this method would allow to correct the chosen mode of treatment to make it more effective. We applied digital PCR method to quantitatively diagnose the disease state of *Felineleukemiavirus* Jarrett, 1964, and *Mycoplasmahaemofelis* Neimark, 2002. The effectiveness of treatment modes was evaluated by comparison of quantitative PCR data. The digital PCR method was applied to quantitatively diagnose Familiar Shar-Pei Fever as diagnostic test that might by useful to breeders.

## Cytogenetics of meiotic cells

### L10

#### Contributions of synaptonemal complex studies to avian cytogenetics

María Inés Pigozzi

*Universidad de Buenos Aires, Facultad de Medicina, Paraguay 2155, Buenos Aires, Argentina* (*mpigozzi@fmed.uba.ar*)

More than 40 years ago, the synaptic nature of the ZW pair in birds was established looking at the synaptonemal complexes in chicken oocytes. The ZW pair forms a synaptonemal complex with lateral elements of unequal lengths. The small pseudoautosomal region in the chicken remains elusive to full sequence characterization, but Z-autosome translocations and recombination nodules analyses at pachytene showed that this region comprises the terminal segment of Zp and Wp. Unlike mammals, the pseudoautosomal regions of birds are extremely variable in size, spanning over 80% of the W chromosome in the primitive ratites. The chromatin of the sex bivalent does not show signs of heteropicnosis during pachytene and, therefore condensation is not required for the pairing of the mainly heterologous Z and W chromosomes of Neognathae. Evidence comprising expression analysis of Z-linked genes and mapping of histone modifications seems to confirm that the chromatin of the ZW pair is not subject to meiosis-specific condensation and silencing in chicken oocytes. Nonetheless, this issue needs more comprehensive analyses using pure fractions of oocytes to measure gene expression of Z-linked genes vs. autosomal genes at specific meiotic stages. Recombination nodules and MLH1 focus mapping in whole pachytene nuclei revealed less interspecific variations of global recombination rates in birds. The mean recombination rate of the birds studied so far is in the range of 1.7–2.6 cM/Mb, while in eutherian mammals it is 0.5–1.1 cM/Mb. The differential organization of the chromatin loops along the synaptonemal complexes in birds and mammals might provide a structural basis for these differences in recombination levels. The changing morphology of the ZW pair in the chicken and other birds with highly differentiated sex chromosomes might be an excellent model to study the dynamics of synaptonemal complex proteins and its relationships with chromatin. Also, the spatial arrangement of recombination nodules particularly found in birds and their relationship with synaptonemal complex components could be analyzed in depth using more recent approaches, such as super-resolution microscopy in combination with immunolocalization.

## O7

### Upgrade sperm FISH analysis of meiotic segregation in a river buffalo bull carrier a rob(1p;18)

Alessandra Iannuzzi^1^, Chiara Di Dio^1^, Angela Perucatti^1^, Anna Caputi Jambrenghi^2^, Francesco Giannico^2^, Alfredo Pauciullo^3^, Pietro Parma^4^, Leopoldo Iannuzzi^1^

**1***Institute for Animal Production System in Mediterranean Environment* (*ISPAAM*), *National Research Council* (*CNR*), *via Argine 1085, Naples, 80147, Italy***2***Department of Animal Productions, Agricultural Faculty of Sciences, University of Bari, Campus Universitario „Ernesto Quagliariello”, 70126 Bari – Italy***3***Dept. Agricultural, Forest and Food Sciences* (*DISAFA*), *University of Turin, Largo Paolo Braccini, 2 – 10095 GRUGLIASCO* (*TO*), *Italy***4***Dep.of Animal Science, Agricultural Faculty of Sciences, Milan, Via Celoria, 2 – 20133 Milan, Italy*

Corresponding author: *Alessandra Iannuzzi* (*alessandra.iannuzzi@cnr.it*)

An upgrade by triple-colour fluorescence *in situ* hybridization (FISH) in River Buffalo sperms was performed on chromosomes involved in the translocation (BBU1 and BBU18) using three different pools of specific bovine BAC (INRA library) probes, mapping in BBU1q, BBU1p and BBU18 (homologous to BTA1, BTA27 and BTA18, respectively). The meiotic segregation pattern was examined in the carrier and in the control counting 2.500 total sperms and 2.500 total motile sperms on both animals. The results revealed different frequencies of normal and chromosomally balanced sperms (alternate group) as follows: 24.1% and 11.4% (total of 35.5%) of total sperms in the carrier, while in the control there were 97.1% and 0.4% (total of 97.8%) respectively; 58.4% and 22.2% (total of 80.6%) of motile sperms in the carrier, while in the control there were 93.6% and 3.3% (total of 96.1%) respectively. These data have shown the increase of percentage in alternate group of motile sperms fraction that represents the real sperm population able to fertilize an oocyte. This result revealed a connection between sperm motility and DNA distribution, underling the importance of Sperm-FISH analysis in reproducers.

**Acknowledgments**

The research was supported by Project PON1_486-GENOBU.

## O8

### The plasticity of meiotic recombination and its implications for mammalian genome evolution

Aurora Ruiz-Herrera^1,2^, Laia Capilla^1^, Covadonga Vara^1,2^, Guillermo Albert-Lizandra^1,2^, Jeremy B. Searle^3^, Jacint Ventura^4^

**1** *Genome Integrity and Instability Group, Institut de Biotecnologia i Biomedicina, Universitat Autònoma de Barcelona, Campus UAB, 08193, Cerdanyola del Vallès, Barcelona, Spain* **2** *Departament de Biologia Cel.lular, Fisiologia i Immunologia, Universitat Autònoma de Barcelona, Campus UAB, 08193, Cerdanyola del Vallès, Barcelona, Spain* **3** *Department of Ecology and Evolution, Corson Hall, Cornell University, Ithaca, NY 14853-2701, USA* **4** *Departament de Biologia Animal, Biologia Vegetal i Ecologia, Universitat Autònoma de Barcelona, Campus UAB, 08193, Cerdanyola del Vallès, Barcelona, Spain*

Corresponding author: *Aurora Ruiz-Herrera* (*aurora.ruizherrera@uab.cat*)

Homologous chromosomes exchange genetic information through recombination, a process that increases genetic diversity and is fundamental to eukaryote organisms with sexual reproduction. A general appreciation of how this genetic variation is generated and maintained across different phylogenetic groups is relevant to our understanding of biodiversity. In an evolutionary framework, meiotic recombination can be modulated by genome reshuffling, thus contributing to chromosomal evolution. However, few empirical data are available regarding the mechanisms by which genome shuffling shapes recombination, especially in mammals. In this context, zones of chromosomal polymorphism of the Western European house mouse, *Musmusculusdomesticus* Linnaeus, 1758, represent a natural model to study such processes. Here we present an empirical study on the impact of chromosomal fusions on genome-wide recombination in mice from wild populations by combining SNPs genotyping data and cytological analysis from meiotic crossovers. We detected a reduction in crossover frequencies in reorganized chromosomes when compared with acrocentric chromosomes. This observation was consistent with the higher genomic divergence detected in pericentromeric regions when compared to telomeric regions being suggestive of differential levels of gene flow associated to chromosomal fusions. Overall, we provide evidence for the contribution of genome reshuffling in modulating recombination landscapes.

## O9

### Interspecies gynogenesis – a way to avoid contamination with radiation induced paternal chromosome fragments

Konrad Ocalewicz^1^, Stefan Dobosz^2^, Krzysztof Jagiełło^1^, Marcin Polonis^1^

**1** *University of Gdansk, Institute of Oceanography, Department of Marine Biology and Ecology, M. Piłsudskiego 46 Av., 81-378 Gdynia, Poland* **2** *Inland Fisheries Institute in Olsztyn, Department of Salmonid Research, 83-330 Żukowo, Poland*

Corresponding author: *Konrad Ocalewicz* (*konrad.ocalewicz@ug.edu.pl*)

Gynogenetic doubled haploids (DHs) as fully inbred fish are useful in developmental biology and aquaculture research. However, it has been noticed that incomplete UV-induced inactivation of the sperm nuclear DNA may result in “contamination” of the gynogenetic specimens with the radiation-induced chromosome fragments. Presumably, such fragments could be eliminated when gametes used for the induced gynogenesis originate from species whose hybrid offspring are lethal due to the genome incompatibility. In the present research gynogenetic development of rainbow trout (*Oncorhynchusmykiss* Walbaum, 1792), brook trout (*Salvelinusfontinalis* Mitchill, 1814) and brown trout (*Salmotrutta* Linnaeus, 1758) was induced using UV irradiated homologous and heterologous spermatozoa. Eggs inseminated with the irradiated sperm were then subjected to high hydrostatic pressure (HHP) shock to inhibit the first cell cleavage and duplicate haploid sets of maternal chromosomes. Produced DH individuals were cytogenetically studied to confirm their ploidy and to detect any karyotypic abnormalities. Survival of gynogenetic DH embryos developing in eggs inseminated with irradiated homologous and heterologous sperm was comparable. Increased mortality among interspecies gynogenotes was observed after hatching. Cytogenetic analysis of DHs confirmed diploid chromosome complements for the studied egg donor species. No chromosome fragments – residues of the UV irradiated paternal genome were found in cells of interspecies gynogenetic DHs. It might be assumed that individuals with UV-irradiated chromosome fragments from heterologous spermatozoa were preferentially lost that could partially explain lower survival of the interspecies gynogenotes when compared to fish that hatched from eggs activated by the irradiated homologous sperm.

## Alphabetical cytogenetics: B-, X-, Y-, W-, Z- etc. chromosome analysis

### L11

#### Dosage-sensitive regulators are preferentially retained on vertebrate Y and W chromosomes

Daniel W. Bellott^1^, Helen Skaletsky^1,3^, Sahin Naqvi^1,2^, Jennifer F. Hughes^1^, David C. Page^1,2,3^

**1** *Whitehead Institute, 455 Main Street, Cambridge, Massachusetts, USA* **2** *Department of Biology Massachusetts Institute of Technology, 77 Massachusetts Avenue, Cambridge, MA, USAn* **3** *Howard Hughes Medical Institute, Whitehead Institute, 455 Main Street, Cambridge, MA, USA*

Corresponding author: *Daniel W. Bellott* (*bellott@wi.mit.edu*)

Across vertebrate lineages, sex chromosomes have repeatedly evolved from ordinary autosomes. In mammals, males are XY and females are XX, while in birds and snakes, males are ZZ and females are ZW. In each of these three lineages, a different set of ancestral autosomes evolved into highly differentiated sex chromosomes. This forms a natural experiment where the autosomes in two lineages serve as a control for the effects of sex chromosome evolution in the third. Sex-specific Y and W chromosomes have experienced extensive genetic decay, and the survivors are enriched for dosage-sensitive regulators of key cellular processes that are broadly expressed throughout adult tissues as well as developmental time. Moreover, survivors of W-decay in both birds and snakes are enriched for human orthologs implicated in congenital disorders caused by heterozygous loss-of-function mutations. We infer that the correct dosage of surviving Y and W genes is likely essential for the survival of the heterogametic sex. We also observe that ancestral differences in dosage sensitivity shaped the evolution of dosage compensation on X and Z chromosomes. Among genes without a surviving Y or W homolog, those that evolved dosage compensation are more sensitive to both under-expression and over-expression than those that did not. Studying the survival of genes on Y and W chromosomes, as well as the evolution of dosage compensation on X and Z chromosomes, may provide insights into key dosage sensitive genes and processes relevant to human health and disease.

## L12

### Evolution of W and Z sex chromosomes in moths and butterflies

František Marec

*Biology Centre CAS, Institute of Entomology, Branišovská 31, 370 05 České Budějovice, Czech Republic* (*marec@entu.cas.cz*)

Moths and butterflies (Lepidoptera) have sex chromosome systems with female heterogamety (WZ/ZZ or derived variants). Results of fluorescence *in situ* hybridization (FISH) with genomic, W-chromosome painting, and BAC (bacterial artificial chromosome) probes along with available sequence information suggest that lepidopteran W chromosomes are almost completely composed of repetitive sequences. The W chromosomes evolve rapidly, and their molecular composition differs considerably even between closely related species, as we have recently shown in the magpie moth, *Abraxasgrossulariata* Linnaeus, 1758, an iconic species in which the female heterogamety was discovered, and its congeneric species *A.sylvata* Scopoli, 1763. On the contrary, Z chromosomes are highly conserved in Lepidoptera, as demonstrated by synteny mapping of Z-linked genes between distant species using BAC-FISH and linkage analyses. The W chromosome is an evolutionary novelty in Lepidoptera, as it is absent in the sister order Trichoptera (caddisflies) and in primitive moths such as Micropterigidae. Our recent data on the W presence/absence in lower Lepidoptera, together with conserved synteny of Z-linked genes, suggest the multiple origin of the W chromosome, although its single origin followed by repeated losses cannot be ruled out. Based on these new data, we have revised the hypothesis on the origin of the W chromosome. However, in a relatively large number of species, the WZ pair was altered by fusion with an autosome pair, resulting in neo-sex chromosomes or multiple sex chromosomes, if the fusion occurred in only one sex chromosome. Extreme cases are wood white butterflies of the genus *Leptidea* Billberg, 1820, with 3-4 W chromosomes and 3-6 Z chromosomes. We have established a battery of genomic tools in *L.juvernica* Williams, 1946, such as the transcriptome sequence, BAC library, and array‐CGH. Here I demonstrate the application of these tools for the identification of sex-linked genes and genomic regions to determine the origin of multiple sex chromosomes in Leptidea species.

## O10

### B chromosomes in the light of functional analyses

Cesar Martins^1^, Rafael T. Nakajima^1^, Bruno E. Fantinnati^1^, Diego F. Marques^1^, Natália B Venturelli^1^, Maryam Jehangir^1^, Erica Ramos^1^, Jordana Oliveira^1^, Syed F. Ahmad^1^, Luiz A. Bovolenta^1^, Adauto L. Cardoso^1^, Guilherme T. Valente^1^

**1***Sao Paulo State University* (*UNESP*), *Institute of Biosciences, Department of Morphology, 18618-689, Botucatu, SP, Brazil***2***Sao Paulo State University* (*UNESP*), *Agronomic Science School, Department of Bioprocess and Biotechnology, 18610-307, Botucatu, SP, Brazil*

Corresponding author: *Cesar Martins* (*cmartins@ibb.unesp.br*)

Supernumerary B chromosomes (Bs) are enigmatic dispensable elements extensively characterized in diverse eukaryote taxa including fungi, animals and plants. The current concept on Bs states they are maintained and propagated by a parasitic-drive mechanism during cell cycle. Nevertheless, the molecular mechanism that governs the drive and other possible effects of B presence is still poorly understood. In this way, we have analyzed B chromosomes of the cichlid fish *Astatotilapialatifasciata* Regan, 1929, in the light of massive DNA and RNA sequencing, FISH-mapping, immunocytogenetics, meiotic transmission, RT-qPCR and epigenomics analysis in order to advance the undrestanding of the B chromosome biological role. We have detected thousands of gene fragments and few largely intact genes in the B chromosome genomic content. Furthermore, differentially expressed transcripts were detected in the presence of B chromosome, they might be transcribed by the B genome or emerge from the A genome under the B chromosome influence. Several of these transcribed sequences, including mRNAs, microRNAs and a possible lncRNA (BncRNA), presented a biased differential expression in the females carrying Bs. In the same way, our results demonstrated that the B chromosome may influence the transcription of DNA modification genes and lncRNAs with consequent epigenetic changes over the cell division regulation machinery. Meiotic analysis indicates a higher drive of Bs in females compared to males in *A.latifasciata* offspring. The integrated view of our data demonstrates B chromosomes can influence cell biology in a complex way, (i) favoring their own maintenance and perpetuation through the cell cycle and also (ii) influencing other important biological features like development and sex determination.

**Acknowledgements**

The work was financially supported by FAPESP 2013/04533-3; 2015/16661-1; CNPq 474684/2013-0;305321/2015-3.

## Clinical cytogenetics

### O11

#### Reciprocal copy number variations and new chromosomal diseases

Igor N. Lebedev

*Research Institute of Medical Genetics, Tomsk National Research Medical Center, RAS, Naberezhnaya. r. Ushaiki, 10, Tomsk, 634050, Russia* (*igor.lebedev@medgenetics.ru*)

Understanding the origin of chromosomal diseases often depends on the development of cytogenetic techniques that provide the precise detection of a wide range of chromosomal abnormalities. At the same time, the progress in investigation the molecular organization and variability of the human genome makes it possible to predict the appearance of recurrent chromosomal aberrations in distinct chromosomal regions. Reciprocal Copy Number Variations (CNVs) is a clear example of uniting theoretical and technological trends in modern human cytogenetics. Genomic sister-disorders were introduced recently as a novel class of chromosomal diseases mediated by duplications versus deletions of the same chromosomal region (Crespi et al. 2009). Currently, more than 60 chromosomal regions in human genome are linked to this type of disease. There is a growing list of reports about new microduplications, or even triplications, quadruplications, etc. for chromosomal regions previously known exclusively for microdeletion syndromes. The comparison of clinical features of patients with reciprocal CNVs provides better understanding of genotype-phenotype correlations for chromosomal diseases. As a result, mirrored, overlapped and unique phenotypes were designated for genomic sister-disorders. It is important, that this type of correlations is associated not only with gene content but also with the effect of chromosome rearrangement. Recently, we demonstrated a significant reduction in the level of CNTN6 expression in neurons differentiated from induced pluripotent stem cells of the patient with 3p26.3 microduplication affecting this single gene only (Gridina et al. 2018). Thus, single gene reciprocal CNVs is another way to clarify the genotype-phenotype correlations for known and new chromosomal diseases. They provide the possibility of phenotypic cleavage of chromosomal syndromes and blur the boundaries between single-gene and chromosomal disorders, creating a genetic continuum for human diseases.

**Acknowledgements**

This study is supported by Russian Science Foundation (14-15-00772).

## O12

### Patterns of gene expression in neurons derived from induced pluripotent stem cells of patients with reciprocal 3p26.3 microdeletion and microduplication

Maria E. Lopatkina^1^, Veniamin S. Fishman^2^, Maria M. Gridina^2^, Nikolay A. Skryabin^1^, Tatyana V. Nikitina^1^, Anna A. Kashevarova^1^, Lyudmila P. Nazarenko^1^, Oleg L. Serov^2^, Igor N. Lebedev^1^

**1** *Research Institute of Medical Genetics, Tomsk National Research Medical Center, RAS, Naberezhnaya. r. Ushaiki, 10, Tomsk, 634050, Russia* **2** *Institute of Cytology and Genetics SB RAS, Lavrentiev 10, Novosibirsk, 630090, Russia*

Corresponding author: *Maria E. Lopatkina* (*lopatkina_maria@mail.ru*)

Microdeletions and microduplications of 3p26.3 were recently shown to be associated with neurodevelopmental disorders. Our goal was to reveal features of gene expression in neurons, derived from induced pluripotent stem cells (iPSC) of patients with intellectual disability and reciprocal aberrations affecting the copy number of *CNTN6* gene only.

Two iPSC clones with deletion, 2 clones with duplication and 3 wild-type clones were differentiated into cortical neurons. The neuronal RNA was examined using SurePrint G3 Human Gene Expression 8×60K Microarray Kit (Agilent Technologies).

It was revealed 676 upregulated and 188 downregulated common genes in neurons with deletion or duplication. Enrichment analysis of upregulated genes revealed their involvement in tissue development and cell adhesion, whereas downregulated genes were involved in the central nervous system and brain development, generation and differentiation of neurons, neurogenesis, axon genesis regulation, sterol and cholesterol biosynthesis. Importantly, 11 genes were upregulated in neurons with deletion but downregulated in duplication. They were involved in regulation of cell and anatomical structure morphogenesis, developmental processes and cell motility, hippocampus and limbic system development. In contrast, 49 genes were upregulated in neurons with duplication but downregulated in cells with deletion. They were involved in chemical synaptic transmission, anterograde trans-synaptic signaling, synaptic and trans-synaptic signaling, regulation of synaptic plasticity and glutamate secretion.

The obtained results provide evidence for a complex pattern of gene expression in iPSC-derived neurons of patients with single-gene genomic sister-disorders.

**Acknowledgements**

This study was supported by Russian Science Foundation (14-15-00772).

## O13

### DNA methylation in inherited copy number variations with incomplete penetrance

Stanislav A. Vasilyev, Nikolay A. Skryabin, Renata R. Savchenko, Ekaterina N. Tolmacheva, Anna A. Kashevarova, Ludmila P. Nazarenko, Elena O. Belyaeva, Igor N. Lebedev

*Research Institute of Medical Genetics, Tomsk National Research Medical Center, RAS, Naberezhnaya. r. Ushaiki, 10, Tomsk, 634050, Russia*

Corresponding author: *Stanislav A. Vasilyev* (*stas.vasilyev@gmail.com*)

Introduction: Chromosomal diseases caused by inherited copy number variations (CNV) are characterized by incomplete penetrance, but its mechanism remains unexplored. In our study, we analyzed differential DNA methylation of both promoter and intragenic CpG in probands with inherited CNV.

Materials and Methods: We have analyzed 15 probands with intellectual disability using aCGH. The presence of CNV in probands, parents and healthy siblings was investigated by realtime PCR. Methylation of promoter and intragenic CpG within a region of inherited CNV was analyzed using targeted bisulfite NGS.

Results: Various CNVs were identified in analyzed probands: dup12q24.12-q24.13 (2 probands from different families, both of maternal origin, ACAD10, ALDH2 and MAPKAPK5 genes); dup3p26.3 of paternal origin (CNTN6 gene); dup1q25.1-q25.2 of paternal origin (ASTN1, TDRD5 etc.); dup5q33.1 on maternal chromosome (AFAP1L1, GRPEL2 and PCYOX1L genes); dup17p13.3 of maternal origin (RPH3AL, VPS53, GEMIN4 etc.); dup18p11.32 of paternal origin (SMCHD1, METTL4 and NDC80 genes); del17q12 (2 probands from different families, both of maternal origin, SLFN11, SLFN13 genes); del9p21.1 (2 probands from different families, both of maternal origin, LINGO2 gene); del7q31.1 (3 probands from 2 different families, both of maternal origin, IMMP2L gene) and del12p11.1 of maternal origin (SYT10 gene). In families with dup18p11.32, dup3p26.3, dup1q25.1-q25.2 and del7q31.1, the methylation index of intragenic CpG was highest in probands and lowest in parents with CNV. Moreover, parents with CNV clustered distinctly from the rest of families.

Conclusion: Intragenic DNA methylation may be the cause of incomplete penetrance of inherited CNV.

**Acknowledgements**

This study was supported by Russian Science Foundation (grant № 16-15-10229).

## Karyotyping and genomics of vertebrate and invertebrate animals

### L13

#### Jurassic spark: Mapping the genomes of birds and other dinosaurs

Darren K. Griffin^1*^, Rebecca E. O’Connor^1^, Michael N. Romanov^1^,Joana Damas^2^, Marta Farré^2^, Henry Martell^1^, Lucas Kiazim^1^, Rebecca Jennings^1^, Anjali Mandawala^3^, Sunitha Joseph^1^, Katie E. Fowler^3^, Eden A. Slack^2^, Emily Allanson^2^, Malcolm Ferguson-Smith^4^, Paul M. Barrett^5^, Nicole Valenzuela^6^, Denis M. Larkin^2^

**1** *School of Biosciences, University of Kent, Canterbury, CT2 7NJ, UK* **2** *Department of Comparative Biomedical Sciences, Royal Veterinary College, University of London, London, UK* **3** *School of Human and Life Sciences, Canterbury Christchurch University, Canterbury, Kent, UK* **4** *Department of Veterinary Medicine, Cambridge University, Cambridge, UK* **5** *Department of Earth Sciences, Natural History Museum, Cromwell Road, London SW7 5BD, UK* **6** *Department of Ecology, Evolution, and Organismal Biology, Iowa State University, USA*

Corresponding author: *Darren K. Griffin* (*d.k.griffin@kent.ac.uk*)

The ultimate aim of a genome assembly is to create a contiguous length of sequence from the p- to q- terminus of each chromosome. Most assemblies are however highly fragmented, limiting their use in studies of gene mapping, phylogenomics and genomic organisation. To overcome these limitations, we developed a novel scaffold-to-chromosome anchoring method combining reference-assisted chromosome assembly (RACA) and fluorescence *in situ* hybridisation (FISH) to position scaffolds from *de novo* genomes onto chromosomes. Using RACA, scaffolds were ordered and orientated into ‘predicted chromosome fragments’ (PCFs) against a reference and outgroup genome. PCFs were verified using PCR prior to FISH mapping. A universal set of FISH probes developed through the selection of conserved regions were then used to map PCFs of peregrine falcon (*Falcoperegrinus* Tunstall, 1771), pigeon (*Columbalivia* Gmelin, 1789), ostrich (*Struthiocamelus* Linnaeus, 1758), saker falcon (*Falcocherrug* Gray, 1834) the budgerigar (*Melopsittacusundulatus* Shaw, 1805). Using this approach, we were able to improve the N50 of genomes seven-fold. Results revealed that Interchromosomal breakpoint regions are limited to regions with low sequence conservation, shedding light on why most avian species have very stable karyotypes.

Our combined FISH and bioinformatics approach represents a step-change in the mapping of genome assemblies, allowing comparative genomic research at a higher resolution than was previously possible. The universal probe set facilitates research into avian karyotype evolution and the role of chromosome rearrangements in adaptation and phenotypic diversity in birds. Indeed, they have been used on over 20 avian species plus non-avian reptiles (including turtles), shedding light into the evolution of dinosaur species. Non-avian dinosaurs remain subjects of intense biological enquiry while pervading popular culture and the creative arts. While organismal studies focus primarily on their morphology, relationships, likely behaviour, and ecology there have been few academic studies that have made extensive extrapolations about the nature of non-avian dinosaur genome structure prior to the emergence of modern birds. We have used multiple avian whole genome sequences assembled at a chromosomal level, to reconstruct the most likely gross genome organization of the overall genome structure of the diapsid ancestor and reconstruct the sequence of inter and intrachromosomal events that most likely occurred along the Archosauromorpha-Archosauria-Avemetatarsalia-Dinosauria-Theropoda-Maniraptora-Avialae lineage from the lepidosauromorph-archosauromorph divergence ~275 million years ago through to extant neornithine birds.

## L14

### Cytogenetic and molecular background of disorders of sex development in domestic mammals

Marek Switonski

*Poznan University of Life Sciences, Wolynska 33, 60-637 Poznan, Poland* (*switonsk@up.poznan.pl*)

Disorders of sex development (DSD) can be caused by sex chromosome abnormalities, single gene mutations, non-genetic factors (e.g. placental anastomoses between heterosexual fetuses) or can have a multifactorial background. It is known that incidence of specific sex chromosome abnormalities is predominant in some species, e.g.: X monosomy in horses, XX/XY leukocyte chimerism in ruminants, XXY trisomy in cats etc. Our recent studies showed that the chimerism is also quite frequent in pigs and occasionally is observed in cats and dogs. Identification of the causative gene mutations in animals with DSD and a normal sex chromosome complement is a challenging task. There are only two monogenic XY DSDs in domestic animals, caused by known mutations. In horses three mutations of androgen receptor (AR) gene, responsible for androgen insensitivity syndrome (AIS), were reported. Secondly, a single mutation of Mullerian inhibiting substance receptor type 2 (MISR2) gene, causing persistent Mullerian duct syndrome (PMDS), is distributed in Miniature schnauzer dogs. In some species (pig, dog, goat, horse) testicular or ovotesticular XX DSD (lack of SRY gene) is quite common. The causative mutation for this DSD, affecting the expression of the FOXL2 crucial for ovarian development, has been identified in goats. Recent studies in dogs and pigs have shown that the mutations may be located near SOX9. Yet knowledge of the genetic background of multifactorial XY DSDs (cryptorchidism and hypospadias) is very scarce. It is known that, in some species the incidence of cryptorchidism is high (about 7% in dogs and horses, or up to 15% in some breeds), while hypospadias is rarely reported. There have been several unsuccessful attempts to find genetic markers associated with predispositions to these DSDs (horse, dog, cat). It can be foreseen that the use of new genomic tools will facilitate identification of new causative gene mutations in the near future.

**Acknowledgements**

The work was financially supported by the National Science Centre, Poland, grant no. 2016/23/B/NZ9/03424.

## O14

### Phylogenetic distribution of the canonical insect TTAGG telomeric repeat within the order Hymenoptera (Insecta)

Vladimir E. Gokhman^1^, Valentina G. Kuznetsova^2^

**1** *Botanical Garden, Moscow State University, Leninskie Gory, Moscow 119234, Russia* **2** *Zoological Institute, Russian Academy of Sciences, 1 Universitetskaya Naberezhnaya, St. Petersburg 199034, Russia*

Corresponding author: *Vladimir E. Gokhman* (*vegokhman@hotmail.com*)

Telomeres are terminal structures of eukaryotic chromosomes that contain repetitive DNA sequences. In the insect order Hymenoptera, both presence and absence of the canonical (TTAGG)n telomeric motif were previously reported. Nevertheless, all those reports investigated telomeric repeats in the suborder Apocrita, whereas DNA sequences of these regions in other hymenopterans were completely unknown. We have recently examined telomeric repeats in two sawfly species of the family Tenthredinidae that belongs to Symphyta, the only other suborder of the Hymenoptera which includes most basal members of the order. In both *Tenthredoomissa* Forster, 1844 and *Taxonusagrorum* Fallen, 1808, telomeres have clearly demonstrated presence of the (TTAGG)n motif, which constitutes the first report of this motif in the Tenthredinidae and Symphyta in general. Although the TTAGG telomeric repeat was long considered characteristic of the Hymenoptera, but, in fact, this repeat was detected until now only on chromosomes of certain aculeate Apocrita, i.e. ants and bees. Moreover, we have subsequently demonstrated absence of the (TTAGG)n telomeric motif in parasitoid Hymenoptera that belong to the same suborder. In addition, it was eventually shown that this motif was absent in all aculeate Hymenoptera except the families Formicidae and Apidae where it apparently reappeared independently. The most comprehensive phylogenetic reconstruction of the order nests the superfamily Tenthredinoidea together with Xyeloidea and Pamphilioidea within a separate clade Eusymphyta which represents a sister group to Unicalcarida, i.e. the remaining Hymenoptera. Furthermore, the (TTAGG)n telomeric motif was also found in many members of the clade Aparaglossata (= non-hymenopteran Holometabola), the sister group to Hymenoptera. Taken together, all these results therefore suggest the ancestral nature of this motif in the order Hymenoptera and its subsequent loss somewhere within Unicalcarida.

**Acknowledgements**

This study was partly supported by research grants no. 18-04-00611 and no. 17-04-00828-a from the Russian Foundation for Basic Research and no. 14-14-00541 from the Russian Science Foundation.

## O15

### Combined analysis of chromosomal and molecular markers reveals cryptic species: karyosystematics of the *Agrodiaetus* Hübner, [1822] blue butterflies

Maria S. Vishnevskaya^1^, Vladimir A. Lukhtanov^1, 2^, Alexandr V. Dantchenko^3^, Alsu F. Saifitdinova^1, 4^

**1** *Saint-Petersburg State University, Universitetskaya emb. 7/9, Saint-Petersburg, 199034, Russia* **2** *Zoological Institute Russian Academy of Sciences, Universitetskaya emb. 1, Saint-Petersburg, 199034, Russia* **3** *Lomonosov Moscow State University, GSP-1, Leninskiye Gory 1/13, Moscow, 119991, Russia* **4** *Saint-Petersburg Scientist’s Union, Universitetskaya emb. 5, Saint-Petersburg, 199034, Russia*

Corresponding author: *Maria S. Vishnevskaya* (*wishm@yandex.ru*)

Butterflies of the subgenus Agrodiaetus Hübner, 1822, genus Polyommatus Latreille, 1804, are the model system in studies of speciation and karyotype evolution. A unique feature of the subgenus is the highest diversity in chromosome numbers in the animal kingdom. In *Agrodiaetus* the number of chromosomes is stable within species and differentiated between species, therefore karyotypes are used for species description, delimitation and identification, although there are exceptions. The use of molecular markers provides an additional information for species delimitation. In our research for testing taxonomic hypotheses we used chromosomal markers in combination with the analysis of two genetically unlinked sequences: fragment of mitochondrial gene *COI* and nuclear spacer *ITS2*. This approach resulted in discovery of five cryptic species inhabiting the Balkan Peninsula – *P.ripartii* Freyer, 1830, *P.nephohiptamenos* Brown et Coutsis, 1978, *P.aroaniensis* Brown, 1976, *P.orphicus* Kolev, 2005 and *P.timfristos* Lukhtanov, Vishnevskaya et Shapoval, 2016 and three cryptic species inhabiting Azerbaijan and Iran – *P.valiabadi* Rose et Schurian, 1977, *P.rjabovianus* Koҫak, 1980 and *P.pseudorjabovi* Lukhtanov, Dantchenko, Vishnevskaya et Saifitdinova, 2015 (Vishnevskaya et al. 2016, Lukhtanov et al. 2015). In general, the data obtained indicate that the genetic and taxonomic diversity of the subgenusAgrodiaetus is significantly higher than it was previously thought.

**Acknowledgements**

The study was supported by the grant N 14-14-00541 from the RSF to the Zoological Institute, RAS. The work was made on the base of “Chromas” Core Facility, Centre for Molecular and Cell Technologies (Saint-Petersburg State University, Research Park), and Department of Karyosystematics (Zoological Institute, RAS).

## Chromatin studies

### L15

#### Structure of holocentric chromosomes

Andreas Houben^1^, Yi-Tsu Kuo^1^, Stefan Heckmann^1^, Andre Marques^2^, Maja Jankowska^1^, Veit Schubert^1^, Jörg Fuchs^1^, Wei Ma^1^, Andrea Pedrosa-Harand^2^, Jiri Macas^3^, Pavel Neumann^3^, Gerhard Wanner^4^

**1***Leibniz Institute of Plant Genetics and Crop Plant Research* (*IPK*) *Gatersleben, Corrensstrassse 3, 06466 Stadt Seeland, Germany***2***Laboratory of Plant Cytogenetics and Evolution, Department of Botany, Federal University of Pernambuco, Brazil***3***Laboratory of Molecular Cytogenetics, Institute of Plant Molecular Biology, Biology Centre AS CR, Ceske Budejovice, Czech Republic***4***Ultrastructural Research, Department Biology I, Biozentrum der Ludwig-Maximillians-Universität München, Großhadernerstr. 2-4, D-82152 Planegg-Martinsried, Germany*

Corresponding author: *Andreas Houben* (*houben@ipk-gatersleben.de*)

The centromere is the chromosome region where microtubules attach and the movement of newly formed chromatids to the daughter cells during mitosis and meiosis occurs. Most organisms contain one single size-restricted centromere per chromosome (monocentric chromosome) visible as a primary constriction during metaphase. It is generally accepted that an independent transition from mono- into holocentromeres occurred in total on at least 13 occasions in eukaryotic lineages (four times in plants). As holocentricity has arisen multiple times during evolution a striking question is whether the organization of centromeres differs in species having independently evolved holocentromeres. The lily *Chionographisjaponica* Maximowicz, 1867 and the grasses *Luzulaelegans* Lowe, 1838 and *Rhynchosporapubera* Boeckeler, 1872 were selected to analyse the organization of their holocentromeres. Strikingly, differences were found in the organization and dynamics of their holocentromeres. Thus, different types of holocentromeres exist.

## O16

### Breakthrough in understanding the phenomenon of lampbrush chromosomes

Alsu F Saifitdinova^1,2,3^

**1***Saint-Petersburg State University, Universitetskaya emb. 7-9, Saint-Petersburg, 199034, Russia***2***International Center of Reproductive Medicine, Komendantskiy av. 53/1, Saint Petersburg 197350, Russia***3***Saint Petersburg Association of Scientists and Scholars, Universitetskaya emb. 5, Saint Petersburg, 199034, Russia* (*saifitdinova@mail.ru*)

Lampbrush chromosomes (LBCs) are specific forms of giant meiotic chromosomes from oocytes of most vertebrates, including birds. They appear as half-bivalent with chromomere-loop organization. The prominent structure of the LBCs loops is maintained by a high density of transcripts. Despite years of research, the reason of such a transcription in meiotic diplotene is unknown. Some studies show that tandem repeats are transcribed in lateral loops, however most transcribed sequences themselves and their biological role still need to be clarified. Contrary to the common opinion about the high speed of transcription on the LBC loops our data indicate a low transcription activity in these areas with blocking release of nascent transcripts from the DNA template. We found various types of repetitive sequences including transposable elements in the composition of the lateral loops on avian LBC whereas euchromatic sequences correspond to the chromomeric regions. Epigenetic markers of chromatin are removed in gametogenesis. Apparently, to regulate the activity of the whole genome in these cells, an alternative way based on the slowdown of transcription is utilized. Stuck transcripts are responsible for the formation of recognizable morphology of lateral loops. Among other things, incomplete transcription in these areas can also increase the level of co-transcriptional mutagenesis therein. It was shown that transcription, along with replication, makes a significant contribution to the level of mutagenesis in the gamete genome. It makes a significant contribution in the maintenance of a stable level of genome variability in populations. This can explain biological significance and widespread presence of LBCs in different groups of animals.

**Acknowledgements**

The study was supported by the Russian Foundation of Basic Research (grant #16-04-01823a). The equipment we used was provided by the “Chromas” Research Resource Center (Saint-Petersburg State University).

## Poster session

### P1

#### Selection of cell lines for the functional validation of 3D genomic interactions by genome editing

Florence Mompart, Yvette Lahbib-Mansais, Anne Calgaro, Hervé Acloque, Martine Bouissou-Matet Yerle

*GenPhySE, University of Toulouse, INRA Occitanie-Toulouse, ENVT, 24 chemin de Borde-rouge 31326 Castanet Tolosan, France*

Corresponding author: *Martine Bouissou-Matet Yerle* (*martine.bouissou-matet@inra.fr*)

Numerous studies have pointed out the major role of 3D nuclear architecture in the regulation of gene expression. Individual chromosomes occupy discrete territories in the nucleus but chromosomal regions often loop out and neighboring chromosomes can intermingle resulting in potential functional contacts between genomic regions from the same or different chromosomes. These cis- and trans-interactions could contribute to gene expression regulation by facilitating the consolidation of co-regulated genes in specific transcription factories. Thus, their dynamic recruitment could result in activation or abatement of gene transcription. In this context, we investigated by means of DNA fluorescence *in situ* hybridization (FISH) the 3D genome organization in porcine fetal muscle cells particularly long-range chromatin interactions implicating the imprinted loci IGF2 (SSC2) and other loci located on different chromosomes (Lahbib-Mansais et al. 2017). We showed that IGF2 alleles associate with the reciprocally imprinted DLK1/MEG3 region (SSC7). We furthermore demonstrated that these trans-interactions preferentially occurred between the expressed alleles. To prove their suggested functional character, we tested cell lines in which we plan to delete the genomic region containing IGF2 to determine if this deletion compromises the occurrence of the interactions. The first step has consisted in analyzing different types of porcine cell lines (4 somatic cell hybrid clones, an iPS cell line, a primary muscle and a permanent cell lines) to determine if they could be a good cell model for this purpose. Both 2D and 3D FISH experiments were performed to verify the presence in these cell lines of: i) the target chromosomes/genes, ii) the genomic interactions between IGF2-DLK1 and IGF2-MEG3. The expression of all target genes was also analyzed. We were able to select one cell line for which we plan to delete IGF2 by CRISPR-Cas9 to determine if this implies the modification of the interactions.

## P2

### Gradual chromosome elimination via micronuclei formation during gametogenesis of di- and triploid interspecies hybrids from *Pelophylaxesculentus* complex

Dmitry V. Dedukh^1^, Sergey S. Rymin^1^, Magdalena Chmielewska^2^, Beata Rozenblut-Kościsty^2^, Krzysztof Kolenda^2^, Mikołaj Kaźmierczak^2^, Maria Ogielska^2^, Alla V. Krasikova^1^

**1** *Department of Cytology and Histology, Faculty of Biological Sciences, Saint-Petersburg State University, 7/9 Universitetskaya emb., 199034 St. Petersburg, Russia* **2** *Department of Evolutionary Biology and Conservation of Vertebrates, Faculty of Biological Sciences, University of Wrocław, Sienkiewicza 21, 50-335, Wrocław, Poland*

Corresponding author: *Dmitry V. Dedukh* (*dmitrov89@yandex.ru*)

Hybrid sterility obstructs reproduction of interspecies hybrids. To cope with sterility some interspecific animal hybrids develop fascinating strategies. One of these strategies, called hybridogenesis, includes selective elimination of one parental genome from germ cells in order to prevent chromosomal conflict in meiosis. To find out how genome elimination is accomplished during hybrid gametogenesis we chose European water frogs complex (*Pelophylaxesculentus* Linnaeus, 1758 complex) as a model. This complex includes two parental species, *P.lessonae* Camerano, 1882 (LL), *P.ridibundus* Pallas, 1771 (RR), and their hybrids, *P.esculentus*. Hybrid frogs exist as a diploid (RL) and a triploid (RRL, LLR) forms which exploit genome elimination for their reproduction. After artificial hybridization experiments we found that diploid males and some females eliminate L genome while the majority of triploids with LLR genotype eliminate R genome. To detect elimination we performed analysis of gonads isolated from hybrid tadpoles with different ploidy. In gonads of hybrid tadpoles, we found micronuclei in cytoplasm of germ cells. Detection of kinetochore proteins using CREST antibodies revealed one signal per each micronucleus indicating that each micronucleus comprises one chromosome. FISH with probe specific to *P.ridibundus* centromeric sequences revealed that triploid LLR hybrids preferentially eliminate R chromosomes eventually forming micronuclei while diploid hybrid frogs preferentially eliminate L chromosomes. We conclude that genome elimination in gonads of diploid and triploid hybrids occurs via gradual elimination of individual chromosomes from one parental genome via micronuclei formation.

**Acknowledgements**

The work was partially performed using experimental equipment of the Research Resource Centers “Environmental Safety Observatory” and “Molecular and Cell Technologies” of St. Petersburg State University.

## P3

### Improving the knowledge of microchromosome repeated sequences in chicken by the sequencing of BAC clones with Pacific Bio technology

Valérie Fillon^1^, Christophe Klopp^2^, Yvette Lahbib-Mansais^1^, Noémie Thebault^1^, Céline Lopez-Roques^3^, Nathalie Rodde^4^, Stéphane Cauet^4^ and Alain Vignal^1^

**1** *GenPhySE, Université de Toulouse, INRA Occitanie-Toulouse, ENVT, 24 chemin de Borde-Rouge, 31326 Castanet Tolosan, France* **2** *Sygenae, INRA Occitanie-Toulouse, 24 chemin de Borde-Rouge, 31326 Castanet Tolosan, France* **3** *Get-Plage, INRA Occitanie-Toulouse, 24 chemin de Borde-Rouge, 31326 Castanet Tolosan, France* **4** *CNRGV, INRA Occitanie-Toulouse, 24 chemin de Borde-Rouge, 31326 Castanet Tolosan, France*

Corresponding author: *Valérie Fillon* (*valerie.fillon@inra.fr*)

Due to the chicken’s (*Gallusgallusdomesticus* Linnaeus, 1758) importance as a model organism and for agriculture, the whole genome assembly was published in 2004. Despite international efforts and many improvements, 138 Mb upon 1.21 Gb are still not assigned to chromosomes (virtual Chromosome Unknown), six microchromosomes remain absent (Gallus_gallus-5.0), and three have a partial coverage. In order to contribute to the sequencing of microchromosomes, we decided to select six BAC clones showing painting signal by FISH on microchromosomes, thus probably containing repeated sequences potentially absent in the assembly.

We performed sequencing with a Pacific Bioscience RSII equipment (P4C6 chemicals). DNA extraction is a crucial step, as both high quantity and quality are needed for Pacbio sequencing. We obtained end sequences for the six BACs before pooling them in the smart cell. As expected, we obtained long reads (9 Kb) and high sequencing depth (953X). Three of these BACs were particularly resistant to sequencing and had to be sequenced separately, demonstrating that some sequence biases could be a possible explanation for the low coverage of microchromosomes in whole genome approaches.

The contigs were established using different assembler: HGAP3, Miniasm, MHAP and Canu. The vector was cleaned, the presence of *Escherichiacoli* Migula, 1895, DNA was filtered and the contigs assigned using the Bac end sequences. The best results were obtained with HGAP3 but Miniasm gave very interesting results with some of the repeated sequences. The alignment against Gallus_gallus-5.0 has allowed to assign some of the Unknown contigs to microchromosomes.

## P4

### The use of interspecific hybrid chromosomes as a tool for precise comparative mapping analysis

Christelle Marrauld^1^, Maria M. Kulak^2^, Svetlana A. Galkina^2^, Alain Vignal^1^, David Gourichon^3^, Francis Minvelle^3^, Valérie Fillon^1^

**1** *GenPhySE, Université de Toulouse, INRA Occitanie-Toulouse, ENVT, 24 chemin de Borde-Rouge, 31326 Castanet Tolosan, France* **2** *Saint-Petersburg University, Universitetskaya nab 7-9, 199034 Saint-Pétersburg, Russia* **3** *PEAT, INRA centre Val de Loire, 37380 Nouzilly, France*

Corresponding author: *Valérie Fillon* (*valerie.fillon@inra.fr*)

The chicken karyotype is considered to closely resemble that of the putative ancestor of modern Aves, presenting most of the characteristics typical of the 10% of avian species karyotyped to date. It possess a large number of chromosomes (2n=78), many of which are indistinguishable ‚microchromosomes’ (30 pairs), females are heterogametic (ZW) whereas males are homogametic (ZZ). Moreover, the knowledge on the chicken genome is wide by comparison with other bird species. That is why the chicken genome is often used as a reference.

Evolutionary studies using zoo-FISH and BAC-FISH have demonstrated birds to have a more stable genome organization compared to mammals, with inter-chromosomal rearrangements corresponding to rarely occurring fusion/fission events. In Galliformes, the karyotype is very stable but generation of high-resolution comparative maps and sequence data have revealed an unexpectedly high number of intra-chromosomal rearrangements if previous cytogenetic data is considered.

We took benefit of F1 interspecific hybrids to develop some precise macrochromosome comparisons between chicken and quail. In the 2000’s in order to study feather coloration genes, a number of cocks (*Gallusgallusdomesticus* Linnaeus, 1758) where mated with japanese quail females (*Coturnixjaponica* Temminck et Schlegel, 1849). A few hybrids were obtained. It was possible to prepare cytogenetic samples from fibroblast cell cultures.

The analysis of the karyotype by classical cytogenetic methods (conventional giemsa staining and G-banding) has allowed the precise characterisation and comparison of macrochromosomes (length, centromere position, banding pattern) for each parental species at the same stage of chromatin condensation. We have also used this unique material for FISH localisation of specific repeated sequences. Thus, we were able to compare the cytogenetic distributions of these sequences in chicken and quail. The use of interspecific hybrid metaphases is a powerful tool for comparative mapping.

## P5

### Chromosome study of a fertile donkey x mare hybrid

Harmonie Barasc, Anne Calgaro, Nicolas Mary, Nathalie Mouney-Bonnet, Clémence Revel, Alain Ducos, Valérie Fillon, Alain Pinton

*GenPhySE, University of Toulouse, INRA Occitanie-Toulouse, ENVT, 24 chemin de Borde-rouge 31326 Castanet Tolosan, France*

Corresponding author: *Anne Calgaro* (*a.seguela@envt.fr*)

The natural mating of a donkey (*Equusasinus* Linnaeus, 1758, 2n = 62) with a mare (*Equuscaballus* Linnaeus, 1758, 2n = 64) produces a male or female hybrid that is usually sterile. To date, very few cases of fertile mules have been reported (Rider et al. 1985, Jones and Johnsen 1985, Rong et al. 1988, Zong and Fan 1989) in contrast to the mullet for which no fertility cases have been reported. In fact, this hybrid belonging to the Equidae family has an intermediate chromosome number between that of the parental species, i.e. 63 chromosomes. This characteristic and the chromosomal differences between the parental species would be responsible for chromosome pairing problems during the first meiotic division and cause meiosis to stop. Nevertheless, a new case of a mule having farrowed a viable male has been identified in Morocco. The karyotype analysis of the latter showed that this hybrid was the product of mating between a mule and a donkey.

## P6

### Genetic analysis of canine mast cell tumors

Hana Hradska, Miluse Vozdova, Svatava Kubickova, Halina Cernohorska, Jan Fröhlich, Jiri Rubes

*Central European Institute of Technology – Veterinary Research Institute, Hudcova 70, 621 00 Brno, Czech Republic*

Corresponding author: *Hana Hradska* (*hradska@vri.cz*)

Being our pets, dogs (*Canisfamiliaris* Linnaeus, 1758) share our environment and develop similar diseases. Cancers cause as much morbidity and mortality in dogs, as they do in humans and are a leading cause of death in dogs over the age of 10. One of the most frequent skin neoplasias in dogs is the collect of mast cell tumors (MCTs) which account for up to 21% of all canine skin tumors. Despite the importance of MCTs in veterinary medicine, little is known about the genetic background of this disease.

To find associations between cytogenetic abnormalities, gene mutational status, histological grade and clinical outcome, we performed the cytogenetic analysis by FISH using a panel of the whole chromosome painting and BAC probes, and molecular analysis of c-kit and TP53 genes in fresh and FFPE samples of canine cutaneous MCTs. The cytogenetic abnormalities varied in the analysed cultured MCTs. However, chromosome 11 abnormalities were observed in all 3 analysed samples. BAC probes for individual canine chromosomes and selected oncogenes are used for detection of a copy number variants in interphase cells of the MCT samples. Mutation analysis of the c-kit (exones 8, 9 and 11), and TP53 (exones 5 and 6) revealed mutations in 18.8% and 6% of the 85 analysed MCT samples. Therefore, we used whole exome sequencing (WES) to search for other molecular aberrations in the coding part of the canine genome which might be causative of the MCT formation. Paired sample approach was used when tumor tissue and blood from the same animal were subjected to WES for elimination of non-causative genetic variants. The data analysis is in progress.

The identification of new MCT-associated genes will improve our understanding of the genetic background of canine MCT, provide data for accurate diagnosis and prognosis and offer a perspective of novel targeted treatment strategies.

## P7

### Analysis of segregation and aneuploidy in a hybrid boar heterozygous carrier of a rob(15;17) by dual-colour-sperm-FISH: preliminary studies

Caputi-Jambrenghi A.^1^, Viviana Genualdo^2^, Francesco Giannico^1^, Bianca Castiglioni^3^, Flavia Pizzi^3^, Donata Marletta^4^, Alessandra Iannuzzi^2^

**1***Department of Animal Productions, Agricultural Faculty of Sciences, University of Bari, Campus Universitario „Ernesto Quagliariello”, 70126 Bari – Italy***2***Institute for Animal Production System in Mediterranean Environment* (*ISPAAM*), *National Research Council* (*CNR*), *via Argine 1085, Naples, 80147, Italy***3***Institute of Agricultural Biology and Biotechnology*, *National Research Council* (*CNR*) , *U.O.S. di Lodi, Via Einstein, 26900 Lodi Lodi, Italy*.

Corresponding author: *Alessandra Iannuzzi* (*alessandra.iannuzzi@cnr.it*)

The Sicily black pig breed is an important autochthonous Italian breed. The animals of the breed are allowed to graze and forage over wide areas (extensive or semi-extensive systems), including woods, and this diet leads to the high quality and flavour of the meat. Crossing between domestic (2n=38) and wild (2n=36) pigs can result in fertile hybrids 2n=37. In fact, during a cytogenetic survey we found two boars with a heterozygous translocation of rob(15;17) (2n=37, XY), normally present in the wild pig. We decided to analyse the sperm segregation, in one of two hybrids, to evaluate the percentage of sperms carrying the translocation. Collected cryopreserved semen samples of 2 boars: one of the hybrid and a control (normal boar) were used for this study. We analysed the sperm segregation and aneuploidy in 2500 sperms, for both samples, by a dual-colour fluorescence *in situ* hybridization (FISH) analysis, using two sets of probe mixtures of specific porcine BAC probes (CHORI library), mapping on SSC15 and SSC17. The preliminary data have shown that the hybrid presents a higher percentage of balanced sperms (alternate II), responsible for transmission of the translocation in progeny, in comparison to the control. These data underline how important it is to conduct cytogenetic studies on reproducers, to preserve autochthonous pure pig breeds and to avoid the selection of hybrids as reproducers.

**Acknowledgements**

This study was supported by CISIA-Gene pig project, National Research Council (CNR) of Rome

## P8

### Genomic analysis on river buffalo (*Bubalusbubalis* Linnaeus, 1758, 2n=50) reared in different conditions: short-and long-term effects on DNA

Alessandra Iannuzzi^1^, Angela Perucatti^1^, Viviana Genualdo^1^, Cristina Rossetti^1^, Domenico Incarnato^1^, Ciro Iorio^1^, Maria Grazia Andreassi^2^, Leopoldo Iannuzzi^1^

**1***Institute for Animal Production System in Mediterranean Environment* (*ISPAAM*), *National Research Council* (*CNR*), *via Argine 1085, Naples, 80147, Italy***2***CNR Institute of Clinical Physiology, c/o Area di Ricerca „S. Cataldo” Via Giuseppe Moruzzi, 1 – 56124 Pisa, Italy*

Corresponding author: *Alessandra Iannuzzi* (*alessandra.iannuzzi@cnr.it*)

DNA damage is the most important factor that induces genome instability and the exposure to exogenous agents (UV light, oxidative stress, chemical mutagens, and radiation) can lead to a variety of modifications of DNA constituents, resulting in genome alterations. The aim of this study is to verify the differences between long-term and short-term DNA damage by different genomic tests: Aberration Chromosomes (CA), Sister Chromatid Exchanges (SCEs) and Cytokinesis-Block Micronucleus (CBMN) for long-term DNA damage and the relative Telomere Lenght (TL), by Monochrome Multiplex Quantitative PCR (MMQPCR) method, for short-term DNA damage. We selected two groups of buffaloes (five for each homogeneous group for age and sex) raised in different environments: urban (group A) and extraurban (group B).

For CA test, we counted 100 cells for a sample with mean values of CA/cell of 0.06±0.26 (A) and 0.05±0.21 (B); for the SCE test, we elaborated 35 cells per sample with SCE-mean values being 9.06±3.73 and 9.02±3.92, in the A and B-groups, respectively. For CBMC test, we counted 500 cells for a sample: mean values of Nuclear Division Index (NDI) was 2.04±0.11 and 1.89±0.04 in the A and B- groups, while the Binucleated Cell Indexes (BCI) were 77.0±7.58 and 75.6±5.41 in the A- and B-groups, respectively. Mean values of the Bi-Nucleated cells with MN (BNMN) and MN for cell Bi-Nucleated they were 1.40±1.52 and 1.80±2.05 in the A- and B-groups, respectively. The TL value (expressed as telomere length relative to a single copy reference gene) was 0.98±0.57 (A) and 1.24±1.07 (B).

For each test no statistical differences were found between the two groups, but it is necessary to study a larger number of animals to validate the results in a better way.

**Acknowledgments**

The research was supported by Project PON1_486- GENOBU

## P9

### First description of karyotypes and localization of ribosomal genes in two sand lances (Perciformes: Ammodytidae); small sand eel (*Ammodytestobianus* Linnaeus, 1758) and great sand eel (*Hyperopluslanceolatus* (Le Sauvage, 1824))

Lech Kirtiklis^1^, Katarzyna Mojsa^2^, Konrad Ocalewicz^2^

**1***Department of Zoology, Faculty of Biology and Biotechnology, University of Warmia and Mazury in Olsztyn, M. Oczapowskiego5, 10-718 Olsztyn, Poland***2***Department of Marine Biology and Ecology, Institute of Oceanography, University of Gdansk, M. Piłsudskiego 46 Av., 81-378 Gdynia, Poland*.

Corresponding author: *Lech Kirtiklis* (*leo@uwm.edu.pl*)

The sand lances also known as sand eels are small fish belonging to family Ammodytidae. These elongated and slender fishes are able to dive into sand to escape predators. Ammodytidae consists of 31 species belonging to seven genera. Despite world-wide distribution, sand lances have rarely been objects of cytogenetic studies. In present research, chromosomes of small sand eel and great sand eel from Gulf of Gdansk (Baltic Sea) have been analysed. Karyotypes of both species were composed of 48 acrocentric chromosomes (FN= 48). Tiny DAPI positive sites were found in the pericentromeric locations of a few sand lances chromosomes. On the contrary, relatively large blocks of NOR-related DAPI-negative chromatin was observed on two chromosomes from small sand eel. Centromeric regions of both fishes studied were resistant to the Hinf I and Dde I restriction endonucleases digestion. Major and minor rDNA sites were observed on separate chromosome pairs in both examined species.

The presented results allowed us to describe the karyotypes of small sand eel and great sand eel, and brought new data regarding organization of the perciform genome related to the largest group of fishes in the world.

## P10

### Polymorphisms of *INSL3* and *ESR1* are not associated with cryptorchidism in dogs

Paulina Krzeminska**^1^**, Maciej Gogulski^2^, Tomasz Nowak^3^, Katarzyna Sochon^4^ and Marek Switonski**^1^**

**1** *Department of Genetics and Animal Breeding, Poznan University of Life Sciences, Wolynska 33, 60-637 Poznan, Poland* **2** *University Centre for Veterinary Medicine, Poznan University of Life Sciences, Szydlowska 43, 60-656 Poznan, Poland* **3** *Department of Animal Reproduction, Poznan University of Life Sciences, Wolynska 35,60-637 Poznan, Poland* **4** *Vetka Veterinary Clinic, Modlinska 65, Warsaw, Poland*

Corresponding author: *Paulina Krzeminska* (*paulina.krzeminska@up.poznan.pl*)

Cryptorchidism, a multifactorial disorder of sex development (DSD), is common in dogs, with an average incidence of 7%. In humans, polymorphisms of 14 genes, including insulin-like protein 3 (*INSL3*) and estrogen receptor 1 (*ESR1*), have been reported as associated with this disorder. It is known that descent of the testes to the scrota depends on the growth of the gubernacula, which is controlled by *INSL3*, while *ESR1* plays an important role in sexual development and reproductive function. We analyzed coding (all exons) and flanking (fragments of introns, as well as 5’- and 3’-flanking regions) sequences of both genes in 19 cryptorchidic dogs, representing 13 breeds. Thirty-four control dogs were also included in the study. Altogether, we sequenced 821 bp of *INSL3* and 5203 bp of *ESR1*. We found 3 SNPs in *INSL3* (g.45071105G>A; g.45071131G>A and g.45071193A>G), while in *ESR1* 1 indel (g.42084005GGGGCA[6_8]) and 6 SNPs (g.42131190T>C; g.42208686A>G; g.42359532G>A; g.42359539T>C; g.42359611A>C and g.42364093G>A) were observed. Two SNPs caused amino acid substitution: g.45071131G>A (Gly2Ser) in *INSL3* and g.42208686A>G (Ile327Val) in *ESR1*. The frequency of the minor variants varied from 0.03 to 0.4, and comparison of their distribution in the cryptorchid and control dogs did not reveal co-segregation with DSD phenotype. In conclusion, our initial study showed that the association of *INSL3* and *ESR1* polymorphism with cryptorchidism in dogs seems to be unlikely.

**Acknowledgements**

The work was financially supported by the Department of Genetics and Animal Breeding, Poznan University of Life Sciences, No. 508.534.01.0.

## P11

### New pericentromeric repeat identified in the genome of japanese quail

Maria M. Kulak^1^, Alexander G. Dyomin^1^, Aleksei S. Komissarov^1^, Valerie Fillon^2^, Alsu F. Saifitdinova^1^, Olga A. Pavlova^1^, Elena R. Gaginskaya^1^, Svetlana A. Galkina^1^

**1** *Saint-Petersburg State University, Universitetskaya emb, 7/9, Saint-Petersburg, 199034, Russia* **2** *GenPhySE, Université de Toulouse, INRA Toulouse-Occitanie, ENVT, 24 chemin de Borde-Rouge, 31326 Castanet Tolosan, France*

Corresponding author: *Maria Kulak* (*ontica@mail.ru*)

Pericentromeres are obligate structural chromosomal parts that guarantee proper chromosome segregation during cell division. Pericentromeric regions of many eukaryotes are enriched in tandem repeats, therefore they are the most difficult genome elements to analyze. The genome of Japanese quail *Coturnixjaponica* Temminck et Schlegel, 1849, the species used in biomedical research and poultry farming, is relatively small (≈1.41 Gb) and packed into 39 chromosome pairs. 41-bp tandem repeats PO41 and BglII are found in centromere regions of CJA4, CJAW and some quail microchromosomes.

Here we report the deciphering of a new tandem repeat CjapSAT found within unassembled Japanese quail short raw reads (GeneBank accession number MH475922). In *C.japonica* genome there are several degenerated and truncated CjapSAT variants with basic repeat unit around 1180 bp and average A+T content of 68%. The CjapSAT repeat is specific to Japanese quail and is not found in any other avian genomes. Meanwhile it has some similarity with centromere repeats of *C.chinensis* Linnaeus, 1766, and *Alectorischukar* Gray, 1830. The presence of motifs homologous to LTR suggests its retroviral origin. FISH with CjapSAT specific probe revealed CjapSAT in pericentromeric heterochromatin on CJA 1-6 and two pairs of microchromosomes, as well as in p- and q-arms of CJAW. At lampbrush stage, the repeat transcribes in long transcription units on lateral loops extending from pericentromeric chromomeres of corresponding autosomes. Despite the species specificity of pericentromeric tandem repeats, their deciphering in sequenced genomes contributes to (1) filling in the gaps of genome assemblies and (2) revealing general patterns of organization of centromeric chromatin.

**Acknowledgements**

The work was done in “Chromas” Research Resource Center and Theodosius Dobzhansky Center for Genome Bioinformatics (SPbSU), with the support of Financial Program 4 of Saint Petersburg State University (#1.40.1625.2017).

## P12

### Loci-specific RNP-rich nuclear domains on lampbrush chromosomes: data pointing at RNA editing

Tatiana V. Kulikova^1^, Lyudmila V. Kazakova^1^, Antonina V. Maslova^1^, Dmitry V. Dedukh^1^, Irina L. Trofimova^1^, Thomas Liehr^2^, Alla V. Krasikova^1^

**1** *Saint Petersburg State University, 7-9 Universitetskaya Emb., St Petersburg 199034, Russia* **2** *Jena University Hospital, Institute of Human Genetics, Am Klinikum 1, 07747 Jena, Germany*

Corresponding author: *Tatiana V. Kulikova* (*t.v.kulikova@gmail.com*)

Ubiquitous RNA-rich nuclear domains, such as nucleolus, paraspeckles and stress-induced domains form as a result of specific loci transcription. Nuclear domains of interphase nuclei disassemble in mitosis and loci of their association are hard to be traced on individual metaphase chromosomes. From this point lampbrush chromosomes (LBCs) – transcriptionally active meiotic bivalents of diplotene oocytes – represent promising model for studying nuclear domains at the loci of their formation. Loci-specific RNP-rich nuclear domains, in particular “lumpy loops” (LLs), are formed on LBCs of the majority studied avian and amphibian species. Earlier we developed an approach for the analysis of tiny chromosome loci using mechanical microdissection of individual chomomeres and lateral loops of LBCs followed by FISH probe generation and next-generation sequencing (Zlotina et al. 2016). Multiple LLs accumulating splicing snRNPs form at specific loci of LBCs of marsh frog Pelophylaxridibundus (Dedukh et al. 2013). We have microdissected four LL-loci on marsh frog LBCs C, D, E and H and generated FISH probes. FISH on metaphase chromosomes revealed single major signals for each probe corresponding to the dissected loci, additional minor signals on other chromosomes and low dispersed signal. DNA+RNA FISH on LBCs revealed multiple signals in chromomeres and RNP-matrix of normal loops indicating the presence of repetitive sequences in the dissected loci. All used probes demonstrated cross-hybridization to the loci of other LLs, indicating that similar sequences may induce LL formation on different chromosomes. Interestingly, all probes hybridized only to the bases of loci-specific structures and adjacent chromomeres but not to the RNP-matrix of LLs. These data suggest that RNA in LLs undergoes promiscuous editing and thus is unable to hybridize with the DNA probe.

**Acknowledgements**

Equipment of SPBURRC “MCT” and “Observatory of environmental safety” was used for this study.

## P13

### A new mutation in the MC1R gene is responsible for golden and cornelian coat colours in the Kurilian Bobtails

Elina Bychkova^1,2^, Anton Markov^2^, Natalia Golubeva^2^, Yulia Golovacheva^2^, Elizaveta Filippova^2^

**1** *Saint-Petersburg State University, Universitetsckaya emb., 7/9, Saint Petersburg, Russia* **2** *Zoogen, Karpovki emb., 5, Saint Petersburg, Russia*

Corresponding author: *Anton Markov* (*anton@zoogen.org*)

Golden colour occurs in many breeds of cats and has various forms of manifestation. The pigments eumelanin and pheomelanin take part in its formation. They are distributed along the length of the hair as two bands, so that pheomelanin is located at the base of the hair, and eumelanin is located on the tip of the hair. The Kurilian Bobtail is a breed of short-tailed cat of Russian origin that has both golden colour, and recessive yellow colour called „cornelian” in its phenotype. Two mutations in the melanocortin 1 receptor (MC1R) gene causing a recessive yellow colour have been described: *e* allele for Amber colour found in Norwegian Forest Cat and *er* allele for Russet colour found in Burmese. Until now, there has been no data for any breed of cats explaining the appearance of the golden colour. As a result of the full sequencing of the coding region of the MC1R gene, a new mutation was identified. We analyzed 68 Kurilian Bobtail cats, of which 12, identified as gold, were found to be heterozygotes, and 16, identified as cornelian, were homozygous for the identified mutation. In the Kurilian Bobtails with other colours this mutation was not found. The gold cats of the other two breeds also did not have a mutation. The obtained data suggest that the mutant allele (*ec*) in the compound with the wild type allele leads to the formation of a golden colour, and in a double dose leads to the total absence of eumelanin in the hair, which manifests itself in the form of the cornelian colour. The results which explain the causes of the formation of the golden colour and the new colour called cornelian in the Kurilian Bobtail cats were obtained for the first time.

## P14

### Neo-X chromosome: independent origin and maintenance of syntenic blocks in highly rearranged karyotypes from genus *Proechimys* J. A. Allen, 1899 (Rodentia-Echimyidae)

Willam Oliveira da Silva^1^, Marlyson J. Rodrigues da Costa^1^, Julio C. Pieczarka^1^, Jorge Rissino^1^, Malcolm A. Ferguson-Smith^2^, Jorge Pereira^2^, Cleusa Y. Nagamachi^1^

**1** *Laboratório de Citogenética, Centro de Estudos Avançados da Biodiversidade, Terreno 11, Parque de Ciência e Tecnologia do Guamá, Avenida Perimetral da Ciência, km 01 – Guamá, 66075-75, Belém – Pará – Brasil* **2** *Department of Veterinary Medicine, Madingley Road, Cambridge, CB3 0ES, United Kingdom*

Corresponding author: *Cleusa Y. Nagamachi* (*cleusanagamachi@gmail.com*)

*Proechimys* J. A. Allen, 1899 genus (Rodentia-Echimyidae) represents one of the most diverse group of Amazonian rodents at genetic and taxonomic levels, with more than 60 karyotypes associated with its 22 species, where Neo-X chromosomes are described. The aim of this study is to investigate the genome modification events in *Proechimysgoeldii* Thomas, 1905 (2n=24♀,25♂/FN=42) and *Proechimysgr.goeldii* (2n=16♀,17♂/FN=16). Whole chromosome probes of *Proechimysroberti* Thomas, 1901 (2n=30/FN=54) were produced and used in comparative chromosomal mapping. Samples of *Proechimysroberti* (PRO, ♂), and *P.goeldii* (PGO, ♂) are from Abaetetuba, Pará, Brazil, and *Proechimysgr.goeldii* (PGG, ♀) from Parintins, Amazonas, Brazil. The chromosomes were obtained by fibroblast cell culture, and submitted to G-banding. Whole chromosome probes were produced by flow cytometry, from sorted chromosomes of PRO, and 18 peaks were identified by same species FISH experiments. Cross-species FISH with PRO probes showed 29 signals on PGO karyotype, and 27 signals on PGG karyotype. The comparative chromosome painting analysis between PGO and PGG karyotypes shows that they differ due to 10 fusion/fission events and one inversion. Eight syntenic blocks are shared between the two taxa (PRO 5/2/3, 6/9/1, 9/5, 6/7, 14/1/4, 8, 11, and 12). Additionally, an independent origin of the Neo-X chromosomes of PGO (PRO 7/*/X) and PGG (PRO X/*/5/2/3) was revealed. Our data indicate a high plasticity of mechanisms that determined the chromosomal evolution of these taxa, and also highlight the potential role of rodents as a model to study the evolution of the sex chromosomes.

## P15

### A case of posterior limb malformation in Montbeliarde cattle

Ioana Nicolae^1^, Daniela Maria Vidmichi^1^, Mihai Bogdan^2^, Cosmin Sonea^3^

**1** *Research & Development Institute for Bovine, Sos.Bucuresti –Ploiesti, km.21, Baloteşti, Ilfov, 077015, Romania* **2** *Experimental Station Moara Domneasca, Kontszbuie street, no. 10, Ganeasa, Ilfov, 077102, Romania* **3** *University of gronomic Sciences and Veterinary Medicine, Bd. Marasti, no. 59, sector 1, Bucharest, 011464, Romania*

Corresponding author: *Ioana Nicolae* (*ioana_nicolae2002@yahoo.com*)

During the routine cytogenetic investigation carried out in 17 Montbeliarde females we identified 5 females with chromosomal instability. One of these females had a calf with posterior limb malformation, characterized by the lack of the left posterior leg. The chromosomal complement of the malformed calf and its mother was severely affected, the number of mono-and bi-chromatid breakages of autosomes and heterosomes, loss of chromosome fragments and gaps being much higher than in other four described cases. The SCEs test has been used and revealed a very high number of sister chromatid exchanges (9-15 SCEs/cell) and particularly, the presence of double interchromatid exchanges in one, two or even three chromosomes of the same metaphase.

To our knowledge, the malformed calf’s mother was treated during the first months of gestation with antibiotic (amoxicillin, gentamicin) for an eye disease (palpebral ulcer) and, in the same period, there was a suspicion of aflatoxin pollution in the farm. Considering all this and knowing that the presence of a toxic chemical agent in the first months of gestation can induce fetal growth and development disorders, the etiology of this congenital malformation could be of a teratogenic nature.

**Acknowledgements**

This study was supported by the Sectorial Project 5.3.3./2015.

## P16

### Chromosome phylogeny of bats of subfamily Micronycterinae (Phyllostomidae) based on multidirectional chromosome painting data

Thayse C.M. Benathar^1^; Cleusa Y. Nagamachi^1^; Patricia C.M. O’Brien^2^; Fengtant Yang^3^; Malcolm A. Ferguson-Smith^2^; Luis R.R. Rodrigues^4^; Julio C. Pieczarka^1^

**1** *Laboratório de Citogenética, Centro de Estudos Avançados da Biodiversidade, Terreno 11, Parque de Ciência e Tecnologia do Guamá, Avenida Perimetral da Ciência, km 01 – Guamá, 66075-75, Belém – Pará – Brasil* **2** *Department of Veterinary Medicine, Madingley Road, Cambridge, CB3 0ES, United Kingdom* **3** *Cytogenetics Facility, Welcome Sanger Institute, Wellcome Genome Campus, Hinxton, Cambridgeshire, CB10 1SA, United Kingdom* **4** *Laboratório de Genética e Biodiversidade, Universidade Federal do Oeste do Pará, Rua Vera Paz, s/n, Bairro Salé, 68035, Santarém, Pará, Brazil*

Corresponding author: *Julio C. Pieczarka* (*juliopieczarka@gmail.com*)

The Neotropical bat species of the subfamily Micronycterinae are classified in two genera, the monotypic *Lampronycteris* Sanborn, 1949, and the diversified *Micronycteris* Gray, 1866, with twelve species. We investigated the karyotype relationships of species of this subfamily using chromosomal banding and multidirectional chromosome painting with whole chromosome probes of *Carolliabrevicauda* Wied-Neuwied, 1821 (CBR) and *Phyllostomushastatus* Pallas, 1767 (PHA). A new cytotype was described for *M.megalotis* Gray, 1842 with 2n = 42, FN = 70, showing a fission of pair 4. Phylogenetic analyses were performed using parsimony (PAUP software) with *Macrotuscalifornicus* Baird, 1858 (Macrotinae) as the outgroup. Few chromosome segments are shared between the *Lampronycteris* and *Micronycteris*, as well as among *Micronycteris* species, showing a high rate of karyotype evolution, with the fixation of a large number of rearrangements (inversions, fusions and fissions). The syntenic association CBR2q/Y2qis a chromosomal signature for Micronycterinae, while the associations PHA11p/3p and PHA4q/3p support the *Micronycteris* monophyly. We compared this chromosome map with 15 other species of Phyllostomidae previously studied to build the phylogeny. Most of the analyzed subfamilies each present a highly derived karyotype and different Phyllostomidae clades have different tendencies of karyotype evolution, including conservative, moderate and intense levels of chromosome reorganization. Our analysis is well supported and congruent with the molecular topologies on the relationship between *Lampronycteris* and *Micronycteris* as sister taxa, as well as the monophyly of *Micronycteris*.

## P17

### Meiotic and gene expression analyses in a case of t(1;15) azoospermic boar

Alain Pinton^1^, Anne Calgaro^1^, Nicolas Mary^1^, Harmonie Barasc^1^, Nathalie. Bonnet^1^, Clémence Revel^1^, Stéphane Ferchaud^2^, Isabelle Raymond Letron^3^, Thomas Faraut^1^, Hervé Acloque^1^, and Alain Ducos^1^

**1***GenPhySE, University of Toulouse, INRA Occitanie-Toulouse, ENVT, 24 chemin de Borde-rouge 31326 Castanet Tolosan, France***2***Génétique, Expérimentation et Système Innovants Poitou Charentes F-17700 Saint-Pierre-d’Amilly, France***3***LabHPEC* (*Laboratoire d’HistoPathologie Expérimentale et Comparée*), *ENVT, Université de Toulouse, France and STROMALab, Université de Toulouse, CNRS ERL5311, EFS, ENVT, Inserm U1031, UPS, Toulouse, France*

Corresponding author: *Alain Pinton* (*a.pinton@envt.fr*)

The systematic cytogenetic screening of young boars carried out for more than 20 years in our laboratory allowed us to accurately estimate the prevalence of balanced structural chromosomal rearrangements in the French pig populations (0.5%). Up to now, more than 39000 boars have been analyzed, and 180 new structural abnormalities have been identified. The most frequent were reciprocal translocations (87%). Contrary to humans, altered semen quality (oligo- or azoospermia) was detected in a few cases only: 4 Y-autosome translocations (Y/1, Y/9, Y/14, Y/16) and one autosome/autosome translocation (1/14).

Here, we report the case of a t(1;15) reciprocal translocation identified in an infertile zoospermic boar. Breakpoints position was determined by mate pair sequencing of microdissected translocated chromosomes. Meiotic pairing and recombination were investigated by immunostaining of the SCP1, SCP3, and MLH1 proteins, and analyzed by classical and super resolution microcopy. Finally, the impact of meiotic pairing impairments on SSC1 and SSC15, as well as SSCX and SSCY gene expression was investigated by qPCR.

Histological analysis revealed a total meiotic arrest at the spermatocyte I stage. The rearrangement was characterized by the translocation of a large part of the SSC15 onto the SSC1, leading to the formation of a tiny derivative chromosome 15. A quadrivalent was observed in 87% of the 113 spermatocytes analyzed, and a trivalent plus univalent in the remaining cells. 40% of the quadrivalents as as well as 33% of the trivalents were associated with the XY body. A γH2AX positive signal on SSC1 or SSC15 chromatin was observed in 87% of the spermatocytes analyzed. These results confirmed the impairment of meiotic process. We will also present on-going results on synaptonemal complex analysis by super-resolution microscopy and the expression of several genes located on SSC1, SSC15, SSCX and SSCY.

## P18

### Cytogenetics: a pertinent tool for analysis of therapeutic mesenchymal stem cells in regenerative medicine

Alain Pinton^1^, Aude Noiret^1,2^, Charlotte Bonnemaison^1,2^, Sophie Ployon^1,2^, Anne Calgaro^1^, Isabelle Raymond-Letron^2^

**1***GenPhySE, University of Toulouse, INRA Occitanie-Toulouse, ENVT, 24 chemin de Borde-rouge 31326 Castanet Tolosan, France***2***LabHPEC* (*Laboratoire d’HistoPathologie Expérimentale et Comparée*), *ENVT, Université de Toulouse, France and STROMALab, Université de Toulouse, CNRS ERL5311, EFS, ENVT, Inserm U1031, UPS, Toulouse, France*.

Corresponding author: *Alain Pinton* (*a.pinton@envt.fr*)

Mesenchymal stem cells (MSCs) are adult stem cells that can be isolated from various human and animal tissue sources. They are multipotent, able to differentiate into osteocytes, adipocytes and chondrocytes lineage and offer large innovative therapeutic potential in regenerative medicine. Many clinical trials are running inhuman medicine, and veterinary use knows a rapid development. However, even if their therapeutic benefits are recognized, the biological mechanisms involved in regeneration are still not fully understood.

MSC, classified as ATMP (Advanced Therapy Medicinal Products), are used either as autologous or allogenic cells, freshly isolated cells or after culture expansion. Major scientific concerns address their tissue action mechanisms. Safety issues are linked to their biodistribution and long term tissue persistence.

To contribute these issues, we develop different cytogenetic tools:

An anti-human FISH probe was created in order to track human MSC in tissues sections of immunocompromised mouse models (efficacy proof of concept, safety studies). The Cot1 probe developed showed its efficiency for the precise tissue tracking of MSCs biodistribution after local or systemic administration.

In the same way, X and Y painting probes were developed to allow, in an immunocompetent canine model, the tracking of canine MSC after administration in an opposite sex host model.

Finally, chromosomal stability of MSCs, in particular in long term culture processes, is still controversial. Structural and numerical chromosomal abnormalities have been reported in Human MSC. Consequently, we aimed to analyze the chromosomal stability of the canine MSCs used in our studies. Preliminary results showed that 10% of these cells are polypoid (4n, 6n) at early passages (P2). Complementary researches are still in progress.

In conclusion, our work shows that cytogenetics provides original and pertinent tools to study MSC biology as well as for safety studies in regenerative medicine approaches for human or veterinary medicine.

## P19

### Molecular-cytogenetic characteristics of the *Gmelinoidesfasciatus* Stebbing, 1899, karyotype

Eugene V. Potapenko, Elena I. Mikhailova, Svetlana A. Galkina, Larisa V. Barabanova

*Department of Genetics and Biotechnology, Saint-Petersburg State University, Universitetskaya emb. 7/9, Saint-Petersburg, 199034, Russia*

Corresponding author: *Eugene V. Potapenko* (*g.r.e.e.d@yandex.ru*)

The Baikal amphipod *Gmelinoidesfasciatus* Stebbing, 1899, is of particular interest for population studies due to its unique adaptation abilities. In the middle of the last century, this indigenous species went through successful acclimation in quite a number of water bodies in Central and North-Western regions of Russia in the course of its direct introduction to enlarge food source for commercial fish. By virtue of the fact that comparatively short period of time elapsed since the start of *G.fasciatus* resettlement and that this species successfully adjusted to new habitat factors, it is possible to suggest that genetic mechanisms would underlay the adaptation process.

Peculiar features of the life cycle of amphipod *G.fasciatus* make it feasible to widely use cytogenetic and molecular-cytogenetic methods for investigation of genome changes at different levels of its organization. Having this objective in mind we analyzed frequencies of chromosomal aberrations in mitotically dividing embryo cells of *G.fasciatus* from different sample collection spots, but did not reveal considerable deviations of the parameter in the studied populations. We assumed that the time interval, starting from the point of introduction of the Baikal amphipod to new water systems, turned out to be sufficient to reach the optimal level.

Using individuals from the studied populations and routine acetoorcein-staining we determined the *G.fasciatus* diploid chromosome number to be equal to 2n=52. It became possible to construct a karyogram although we could not reveal specific morphological characteristics that would make every individual chromosome pair identification possible. Even so it became obvious that several pairs could be distinguished as being longer and having varied but similar within pairs chromatin appearance. DAPI-staining revealed A-T rich pericentromeric regions of mitotic chromosomes. FISH of telomeric repeats (TTAGG)n detected hybridization sites at chromosome ends, however some interstitial locations have been revealed as well. Hybridization of 18S rDNA molecular probe with *G.fasciatus* chromosomes manifested 4 distinct signals on 2 pairs of chromosomes in most of the nuclei. At the same time we have registered the presence of more than 4 (from 5 to 8) hybridization signals in several nuclei which indicates the variability of ribosomal cluster numbers in amphipod karyotype. Sequencing of 18S rRNA gene in particular conserved and variable fragments amplified using DNA of amphipod from indigenous Baikal population and those from different collection spots in the Gulf of Finland as well as from the Lake Ladoga showed no differences between specimens. Prospects of devising more molecular markers and *in situ* hybridization probes for the sake of disclosing more variation between *G.fasciatus* populations will be discussed with a view to getting insight into mechanisms of adaptation processes.

**Acknowledgements**

The research was partially supported by the grants, RFBR 15-29-02526 and NSH 9513.2016.4, and performed at the Research park of St.-Petersburg State University Centers for Molecular and Cell Technologies and “Chromas”.

## P20

### Multiple highly amplified NORs co-localized with telomeric sequences in the parthenogenetic hybrid *Bacilluswhitei* Nascetti et Bullini, 1982, (InsectaPhasmatodea)

Susanna Salvadori^1^, Elisabetta Coluccia^1^, Federica Deidda^1^, Michela Sibiriu^1^, Erica Costa^1^, Anna Maria Deiana^1^, Valerio Scali^2^

**1***Universiy of Cagliari Department of Life and Environmental Sciences* (*Università of Cagliari, Dipartimento di Scienze della Vita e dell’Ambiente*), *Via T. Fiorelli 1, 09126 Cagliari, Italia***2***Universiy of Bologna Department of BiGeA* (*Università di Bologna, Dipartimento BiGeA*), *Via Selmi 3, 40139 Bologna, Italia*

Corresponding author: *Susanna Salvadori* (*salvador@unica.it*)

Stick insects (Insecta, Phasmatodea) are a very interesting group for their reproductive biology. In fact most of them present bisexual reproduction but the lytokous parthenogenesis, hybridogenesis and androgenesisare are widespread among them. In fact, about 20% of the 3000 species are obligatory parthenogenetic, often as a consequence of hybridization between species. (reviewed in Scali 2009; Milani et al. 2015). Previous karyological investigations had evidenced extensive numerical and structural chromosome re-patterning in hybrid parthenogenetic taxa, leading to a variety of cytotypes, and a wide array of Ag-NOR bearing chromosomes in different species and even populations within species (Manaresi et al. 1992; 1993). In this work we analyzed by dual-FISH of 28S rDNA and telomeric pentameric sequences (TTAGG)n the karyological features in two populations of the parthenogenetic the lytokous *Bacilluswhitei* Nascetti et Bullini, 1982 (2n=35 XX). This is a diploid hybrid, endemic of Sicily (Italy), between *B.rossius* (2n=36/35; XX/X0) and *B.grandii* Nascetti et Bullini, 1982 (2n=34/33, XX/X0) (Manaresi et al. 1992). Our results showed differences between the two populations in karyotype features such as chromosome rearrangements and the number/position of the highly amplified NORs. Furthermore, we constantly found a co-localization between rDNA and highly amplified telomeric sequences, as first evidenced in phasmid taxa of the genus *Leptynia* Pantel, 1890 (Scali et al. 2016). A similar interspersion has been also pointed out in five additional species of Phasmatodea belonging to distantly related genera (Liehr et al. 2017). The sharing of this type of amplification-interspersion of NOR and telomeric sequences in all phasmid species investigated by FISH strongly supports our opinion that their relationship could be not a casual one and also supports the early hypothesis that the NOR/telomere interspersion might constitute a hot spot of recombination (Salvadori et al. 1995), as actually shown by the variable NOR numbers and positions in *B.whitei*.

## P21

### Optimizating the analysis of porcine adipose tissue derived mesenchymal stem cells karyotyping

Raul Sánchez-Sánchez, Paloma De la Cruz Vigo, Ernesto Gómez Fidalgo, Sonia S. Pérez-Garnelo, Alexandra Calle, Miguel A. Ramirez, Mercedes Martin-Lluch

*INIA. Avda. Puerta de Hierro Km. 5,9. Madrid, Spain*

Corresponding author: *Raul Sánchez-Sánchez* (*raulss@inia.es*)

Cytogenetic studies are a reliable indicator of genetic stability of Adipose tissue-derived mesenchymal stemcells (ASCs), since these cells can lead to an accumulation of genetic and epigenetic alterations during their growing in *in vitro* culture. In particular, karyotype analys is isessential for the identification of both, numerical and structural chromosomal abnormalities.

There are several reasons for obtaining porcine ASCs. First, adipose tissue is abundant and easily accesible, second because pigs are used as a model for preclinical studies in human regenerative medicine, and for last, it may be interesting to store criopreserved porcine ASCs as a reserve of genetic material from pigs with chromosomal rearrangements for future research.

The aim of this study was to optimize the karyotyping method used in porcine ASCs to increase the number and the quality of obtained methaphases. Mesenchymal cells of abdominal adipose tissue were obtained from a boar with a chromosomal alteration (xy/xx mosaicism). Several tests were carried out on the established protocol for fibroblasts [The AGT CytogeneticLaboratory Manual], with modifications in colchicine and hypotonic solution concentrations, exposure times and fixation solution composition. Our results showed that metaphases quality was higher using porcine ASCs incubated with a final dilution of 0.1µl/ml colchicine for 18 hours at 37 °C, treated with prewarmed hypotonic lysis solution (0.054M KCl) at room temperature for a time greater than 10 min. and fixed in methanol:aceticacid (3:1,v/v) solution. The adjustment to colchicine and hypotonic solution exposure is a critical step and thus, shorter periods of colchicine exposure were not enough to obtain a reasonable number of metaphases.On the other hand, higher exposure times and lower KCl concentrations provided better dispersion of methaphase chromosomes.

For that, standardization of karyotyping test is a crucial step to correctly interprete the results in porcine ASCs.

## P22

### Knockout of *ADAMTS1* gene by CRISPR/Cas9 affect the micronucleus frequency but not the DNA repair signaling in HeLa cell line

Renata R. Savchenko^1^, Stanislav A. Vasilyev^1^, Veniamin S. Fishman^2^, Anastasiya A. Murashkina^3^, AnastasiyaV. Dorofeeva^4^, Igor N. Lebedev^1^

**1** *Research Institute of Medical Genetics, Tomsk National Research Medical Center of the Russian Academy of Sciences, 10 Nab. Ushaiki, Tomsk, 634050, Russia* **2** *Institute of Cytology and Genetics of Siberian Branch of the Russian Academy of Sciences, 10 Lavrentyeva Prospect, Novosibirsk, 630090, Russia* **3** *National Research Tomsk State University, 36 Lenin Avenue, Tomsk, Russia* **4** *Siberian State Medical University, 2 Moskovsky trakt, Tomsk, Russia*

Corresponding author: *Renata R. Savchenko* (*savchenko_renata@mail.ru*)

Nowadays different DNA repair mechanisms are known, but the role of other indirect participants of the DNA damage response in maintaining the stability of the genome is insufficiently explored. Our previous transcriptome-wide experiments allowed us to identify differentially expressed genes in lymphocytes of individuals with different levels of repair of double-strand DNA breaks. The expression of several genes was correlated with the level of spontaneous γH2AX foci and the frequency of radiation-induced centromere-negative micronuclei not only in lymphocytes, but also in another cell type, namely human placental fibroblasts. The aim of this study was to analyze the effects of knockout of these genes on the DNA repair and genome integrity in model system *in vitro*.

We created HeLa cell lines with knockout of ADAMTS1, RBFOX2 and THBS1 genes using CRISPR/Cas9 technology. Level of spontaneous γH2AX and 53BP1 foci was assessed as a marker of signaling associated with DNA double-strand breaks. FISH-based micronucleus test was used to analyze the level of chromosome damage.

Knockout of all analyzed genes did not affect significantly the level of spontaneous γH2AX and 53BP1 foci. However, a significant increase in the level of micronuclei was observed inADAMTS1knockout cell line (21.7 ± 7.5 ‰) in comparison with the intact HeLa (5.0 ± 1.0 ‰, p = 0.019). We suppose that ADAMTS1can act as an indirect participant of mechanisms of genome integrity maintenance without affecting DNA double-strand break repair signaling.

**Acknowledgements**

This study was supported by Grant of the President of Russian Federation MK-5944.2018.4.

## P23

### Identification of SNP polymorphism in the *growth hormone* and *myostatin* genes of the two Polish geese breeds

Grzegorz Smołucha^1^, Anna Kozubska-Sobocińska^1^, Anna Koseniuk^1^, Mirosław Lisowski^2^, Bartosz Grajewski^3^, Igor Zubrzycki^1^

**1** *National Research Institute of Animal Production, Krakowska 1, 32-020 Balice n. Cracow; Department of Animal Genomics and Molecular Biology. Poland* **2** *National Research Institute of Animal Production, Krakowska 1, 32-020 Balice n. Cracow; Department of Animal Reproduction Biotechnology, Poland* **3** *National Research Institute of Animal Production, Experimental Station in KołudaWielka, Waterfowl Genetic Resources Station in Dworzyska, 62-023 Dworzyska Poland*

Corresponding author: *Grzegorz Smołucha* (*grzegorz.smolucha@izoo.krakow.pl*)

Advances in molecular genetics of livestock animals allowed for the identification of genes which are responsible for production traits like lean body mass, as well as gene polymorphisms. So far, several genes were used as candidate genes for improvement of productive performance in animals. Among those, the growth hormone (GH) and myostatin (MSTN) play the paramount role. Growth hormone promotes muscle growth, bone formation and regulation of fat content. Myostatin is a major regulator of myogenesis expressed predominantly in skeletal muscle and a negative regulator of skeletal muscle growth and development. To determine the relationship between polymorphism and production performance rendered by the genes of growth hormone and myostatin the Single Nucleotide Polymorphism of the respective genes sequences was studied. The two breeds of Polish goose, i.e., Kielecka and Landes were selected for the study. An analysis of the GH sequence revealed the presence of three single nucleotide polymorphisms (SNP): nonsynonymous (V → A) SNPc.128C>T described previously and associated with the production features of many different breeds in China, and g.240108A> G and g.240247A> G of currently unknown phenotypic and physiological function. The two SNPs were also found in MSTN gene (exon 3): c.1124C>A resulted in amino acid exchange to stop codon and c.1231C>T in the 3`untranslated region.

## P24

### The role of nuclear architecture in regulating *PPARG* gene expression during porcine adipogenesis

Joanna M. Stachecka^1^, Joanna Nowacka-Woszuk^1^, Paweł A. Kolodziejski^2^, Izabela M. Szczerbal^1^

**1** *Department of Genetics and Animal Breeding, 33 Wolynska Street, 60-637 Poznan, Poland* **2** *Department of Animal Physiology and Biochemistry, Poznan University of Life Sciences, 35 Wolynska Street, 60-637 Poznan, Poland*

Corresponding author: *Joanna M. Stachecka* (*jstachec@up.poznan.pl*)

Adipogenesis in pig, as in other organisms, is mediated by a key transcription factor, peroxisome proliferator-activated receptor gamma, encoded by the *PPARG* gene located on porcine chromosome 13. Previous studies have shown that, during adipocyte differentiation, *PPARG* is repositioned from nuclear periphery to nuclear interior without relocation of chromosome territory. Here we examine in detail how changes in nuclear positioning of the *PPARG* gene affect its expression. An established *in vitro* adipocyte differentiation system from mesenchymal stem cells derived from bone marrow and adipose tissue was used. Differentiation was monitored for seven days and cells were examined using a 3D DNA/RNA/immuno-FISH approach. *PPARG* transcript level was measured by real-time PCR, and PPARG activity was detected by colorimetric assay. Changes in the nuclear location of the *PPARG* gene were seen when we compared undifferentiated mesenchymal stem cells with mature adipocytes. Also, the two *PPARG* alleles had different nuclear locations when measured in relation to the nuclear lamina. The RNA–DNA FISH approach has shown that differences in primary transcript production depend on the allele’s nuclear positioning, with transcriptionally active alleles preferentially occupying the central part of the nucleus. The number of *PPARG* transcripts evaluated by RNA-FISH on the single-cell level corresponded to the transcript level and protein activity evaluated by the molecular approach. However, the advantage of FISH-based techniques was that temporal changes in mRNA production dynamics could be detected. Our study confirms the importance of nuclear architecture in regulating gene expression and thus in the establishment of adipose cell fate.

**Acknowledgements**

The work was financially supported by the National Science Center, Poland, grant no. 2012/07/E/NZ9/02573.

## P25

### Expression of key genes involved in DNA methylation during *in vitro* differentiation of porcine mesenchymal stem cells (MSCs) into adipocytes

Joanna M. Stachecka, Weronika Lemanska, Izabela M. Szczerbal

*Department of Genetics and Animal Breeding, Poznan University of Life Sciences, 33 Wolynska Street, 60-637 Poznan, Poland*

Corresponding author: *Joanna M. Stachecka* (*jstachec@up.poznan.pl*)

DNA methylation plays an important role in regulating gene expression and is essential for cell differentiation, including adipogenesis. The proper course of DNA methylation depends on the functioning of the major components of the DNA methylation machinery, including DNA methyltransferase enzymes and proteins that bind specifically to methylated DNA. It has been hypothesized that changes in DNA methylation during porcine adipocyte differentiation are related to changes in the transcription levels of genes crucial to the DNA methylation machinery. We selected five genes (*DNMT1*, *MeCP2*, *MBD1*, *MBD3* and *UHRF1*) and analyzed their expression using real-time PCR and immunofluorescence in undifferentiated mesenchymal stem cells derived from bone marrow (BM-MSC) and adipose tissue (AD-MSC) during the course of adipocyte differentiation. The transcript level of the *MeCP2* gene was found to be lower in AD-MSC differentiation system than in BM-MSC. At the initial days of adipogenesis, this gene was upregulated and its transcript level decreased during the terminal stages of differentiation. A low number of nuclei positive for the MeCP2 protein were also observed on day 7 of differentiation. The *DNMT1* gene was downregulated during the following days of adipocyte differentiation in both the studied systems. In case of *MBD1, MBD3*, and *UHRF1* genes we observed stable transcript levels during adipogenesis. The results indicate the significance of the DNA methylation machinery for proper adipocyte differentiation in pigs.

**Acknowledgements**

The work was financially supported by the National Science Center, Poland, grant no. 2012/07/E/NZ9/02573.

## P26

### Association of a known G-insertion upstream of *SOX9* with XX disorder of sex development in dogs is doubtful

J. Nowacka-Woszuk^1^, I. Szczerbal^1^, M. Stachowiak^1^, S. Dzimira^2^, W. Nizanski^2^, R. Payan-Careira^3^, E. Wydooghe^4^, T. Nowak^1^, K. Blasiak^5^, A. Solecka^6^, M. Switonski^1^

**1***Poznan University of Life Sciences, Wolynska 33, 60-637 Poznan, Poland***2***Wroclaw University of Environmental and Life Sciences, C.K. Norwida 31*, 50-375 Wrocław, Poland **3***Wroclaw University of Environmental and Life Sciences, Pl. Grunwaldzki 49*, 50-366 Wroclaw, Poland **4***Universidade de Trás-os-Montes e Alto Douro, Quinta de Prados, Apartado 1013, 5001-801 Vila Real, Portugal***5***Ghent University, Salisburylaan 133, 9820 Merelbeke, Belgium***6***VetMedica Veterinary Clinic, Zamenhofa 37, 64-100 Leszno, Poland***7***Medwetar Veterinary Clinic, Zeromskiego 105, 64-920 Pila, Poland*

Corresponding author: *Marek Switonski* (*switonsk@up.poznan.pl*)

Testicular or ovotesticular XX, SRY-negative (XX) disorder of sex development (DSD) is commonly diagnosed in numerous dog breeds, but its molecular background remains unclear. In a candidate region harboring SOX9, duplication of this gene, CNV, as well as an indel (–/G, rs852549625) located upstream of SOX9 has been suggested as associated with this DSD. We analyzed 32 unrelated XX (SRY-negative) DSD dogs, including 18 new cases, and 31 control females. We also studied two families in which 3 XX DSD cases had been identified. Cytogenetic analysis, molecular detection of the Y-linked genes (SRY and ZFY), and histological studies revealed, that among unrelated cases, ten were testicular XX DSD and eight were ovotesticular XX DSD, while the histology of the gonads was unknown in 14 cases. Sanger sequencing of the fragment (839 bp) harboring the G-insertion was performed. Apart from the analyzed indel, we identified five known and one unknown SNPs. In a cohort of unrelated XX DSD cases, the following genotypes were observed for the indel: G/G (n = 2), -/G (n = 8) and -/- (n = 22), while in control females, the number of genotypes were 0, 7, and 24, respectively. In a Pug family, two XX DSD siblings and their father had the G/G genotype, while the mother was -/G. In a Cane Corso family, two offspring were heterozygotes, including an XX DSD case, and the parents were G/G (mother) and -/G (father). We conclude that G-insertion upstream of the SOX9 gene is not associated with the affected phenotype in the studied cases.

**Acknowledgements**

The work was finencially supported by National Science Centre, Poland, grant no. 2016/23/B/NZ9/03424.

## P27

### Detection and quantification of leukocyte chimerism (XX/XY) using FISH and digital droplet PCR (ddPCR) in the offspring of highly prolific sows

Izabela Szczerbal^1^, Joanna Nowacka-Woszuk^1^, Stanislaw Dzimira^2^, Anna Alama^3^, Pawel Iskrzak^3^, Marek Switonski^1^

**1** *Department of Genetics and Animal Breeding, Poznan University of Life Sciences, Wolynska 33, 60-637 Poznan, Poland* **2** *Department of Pathology, Wroclaw University of Environmental and Life Sciences, C.K. Norwida 31, 50-375 Wroclaw, Poland* **3** *AgriPlus Poznan, Marcelinska 92/94, 60-324 Poznan, Poland*

Corresponding author: *Izabela Szczerbal* (*izabel@up.poznan.pl*)

Disorders of sex development (DSD) are a significant problem in pig production, since they lead to sterility and may affect meat quality through the presence of testicular tissue in animals of female karyotype. So far, testicular XX DSD (*SRY*-negative) has been recognized as the most common type of this disorder in pigs. In this study, we performed a complex cytogenetic and molecular analysis of 28 pigs with ambiguous external genitalia identified on a large commercial farm with highly prolific sows (~17 piglets/litter). We used standard Giemsa staining and FISH technique with X chromosome-specific probe (BAC clone CH242-156O11 for pseudoautosomal region). The *SRY*, *ZFX*, and *ZFY* genes were detected in blood samples, and in 20 cases in hair follicles, to exclude or confirm whole-body chimerism. Moreover, digital droplet PCR (ddPCR) was used to estimate X and Y chromosome copy numbers, based on copies of the *AMELX* and *AMELY* genes, in order to detect leukocyte chimerism. Among the examined animals, 20 were diagnosed as freemartins due to the presence of leukocyte chimerism (38,XX/38,XY), six had testicular XY DSD and two had XX DSD. The percentage of XX and XY cell lines in the freemartins varied over a wide range. The ddPCR approach turned out to be enough sensitive to detect the cell lines occurring at low frequency. Our study showed that high prolificacy is associated with the occurrence of freemartinism. Although the FISH approach is recommended as a gold standard in the cytogenetic diagnosis of freemartinism, the method is labor-intensive and time-consuming. We thus recommend ddPCR for rapid and reliable detection of the chimerism.

**Acknowledgements**

The work was financially supported the Department of Genetics and Animal Breeding, Poznan University of Life Sciences, No. 508.534.00.

## P28

### mtDNA variation of troglophilic crickets (Myrmecophilidae: Eremogryllodes), across Zagros Mountains and south of Iran

Mohadeseh S. Tahami^1^, Mina Hojat Ansari^2^, Saber Sadeghi^1^, Alsu F. Saifitdinova^3^

**1** *Entomology Research Laboratory, Biology Department, Adabiat St., Shiraz University, Shiraz, Iran, 7146713565* **2** *Gastroenterohepatology Research Center, Shiraz University of Medical Sciences, Khalili St., Shiraz, Iran, 71935-1311* **3** *Chromas Research Resource Center, Saint Petersburg State University, Oranienbaumskoye sh. 2, 198504, Saint Petersburg, Russia*

Corresponding author: *Mohadeseh S. Tahami* (*tahami.m@hotmail.com*)

Several new species from the genus *Eremogryllodes* Chopard, 1929 (Insecta: Orthoptera: Myrmecophilidae) inhabiting a number of caves located across Zagros Mountains and south of Iran, have been recently described based on morphology by the first author (Tahami et al. 2017). These species are *E.bifurcates* Tahami et Gorochov, 2017, *E.dilutes* Tahami et Gorochov, 2017, *E.iranicus* Tahami et Gorochov, 2017 and *E.persicus* Tahami et Gorochov, 2017, and a number of subspecies. To our knowledge, the populations of those species spend most of their life span inside caves, as we have not encountered them outside the cave environment during our extensive surveys in Iran. The described species are morphologically very similar, which hampers the species identification. Therefore, molecular studies are essential to confirm their status. The absence of the distinct morphological characters, applicable for species separation, and the considerable genetic isolation can lead to the formation of the cryptic species. Therefore, our work has two main aims: (1) to test whether the morphological characters, published in Tahami et al. (2017), correlate with the molecular evidences; (2) to test for the presence of the cryptic species. To this extent, here we provided the molecular phylogenetic analysis based on 16S rDNA of the recently described species of *Eremogryllodes* for the first time. Overall, we included 37 specimens collected in 24 caves. Phylogenetic analyses, using RAxML and Bayesian inference methods, revealed four well-supported clusters. The resulting tree also shows one specimen, which does not cluster with the others, and can represent the potential cryptic species. This topology does not corroborate morphological taxonomy. The first cluster corresponds to *E.bifurcatus*. This also corresponds to the geographic distribution, as the specimens of this species were collected from the caves located in the north of Zagros Mts, whereas all other species were mostly collected in the middle and south of the region. The second cluster includes four specimens from *E.persicus*, however, other specimens from this species are intermixed with *E.iranicus* and *E.dilutus* within the third and fourth clusters. Two latter species are also intermixed with each other. Overall, the current investigation is not congruent with three previously described species, and indicates that the morphological characters, although valuable, but cannot always be applicable to species identification in this genus by their own. However, further molecular work using more genes and more specimens from the same region is needed to confirm the status of the clusters provided by 16S and presence of the cryptic species.

**Acknowledgements**

The work was done in Chromas Core Facility Center of Saint-Petersburg State University.

## P29

### Some unusual properties of constitutive heterochromatic regions of chromosome from human extraembryonic tissues

Irina L. Trofimova^1,2,3^, Natella I. Enukashvily ^4^, Mikhail A. Dobrynin^5^, Tatyana V. Kuznetzova^6^

**1** *Federal State Budgetary Institution V.A. Almazov Federal North-West Medical Research Centre of the Ministry of Health of the Russian Federation, Akkuratova street 2, 197341, Saint-Petersburg, Russia* **2** *International centre of reproductive medicine, Komendantskij prospect, 53k, 197350, Saint-Petersburg, Russia* **3** *Federal State Budget military educational institution of higher professional education “Military Medical Academy named after S.M. Kirov” under the Ministry of Defense of the Russian Federation, 6 Lebedeva street, St. Petersburg, 194044, Russia* **4** *Institute of Cytology of the Russian Academy of Sciences, Saint-Petersburg, Tikhoretsky ave. 4, 194064, Saint-Petersburg, Russia* **5** *Saint Petersburg State University, Universitetskaya nab. 7/9, 199034, Saint Petersburg, Russia* **6** *Federal State Budgetary Scientific Institution “The Research Institute of Obstetrics, Gynecology and Reproductology named after D. O. Ott”, Mendeleevskaya street, 199034, Saint-Petersburg, Russia*

Corresponding author: *Irina L. Trofimova* (*il_trofimova@list.ru*)

Satellite DNA forms large arrays within heterochromatic regions of chromosomes and constitutes an essential part of the “non-coding landscape” of eukaryotic genomes. Satellite DNA of constitutive heterochromatin regions (CHRs) of autosomes 1, 9, 16 of human is of special interest and its functional value is discussed. These CHRs in chromosomes from cytotrophoblasts of chorion villi are characterized by decondensation, early replication, hypomethylation and DNAse 1 hypersensitivity.

We studied peculiarities of CHRs on direct and semi-direct slides from chorionic villi. After standard acridine orange (AO) staining of untreated direct chromosomal preparations CHRs manifest unusually bright red fluorescence in 1qh, 9qh, 16qh as typical for single-stranded DNA and RNA. The nature of this fluorescence was studied in direct chromosomal preparations pretreated with different enzymes (RNase A, RNase H, DNase I, DNA ligase T4) followed by AO staining. Results of this work show that ssRNA and DNA*RNA hybrids are present in CHR of chromosome 1 (1q12) on fixed chromosomes.

We studied in details nuclear position and transcriptional activity of satellite 3 of CHR of 1q12. Our 3D-FISH results showed a significant repositioning of 1q12 towards the centre of the nucleus and near chromocenter in chorionic villi sample from early pregnancy (4-5 week). We found no changes in the position of 1q12 in chorionic villi from 5-6 week to 36 week pregnancy. Almost all FISH-signals were closer to the nuclear periphery and chromocenter. RT-PCR results showed polyadenilated non-coding RNAs of 1q12 in chorion on 6-15 weeks of gestation. The choice of the chain for transcription depends on gestation age.

The results confirm unusual conformational packaging of CHRs which can be associated with transcriptional activity of satellite DNA of 1qh in chorionic villi cells during early embryogenesis.

**Acknowledgements**

This research was supported by Russian Foundation for Basic Research (grant #18-34-00279).

## P30

### Two cases of equine X chromosome monosomy mosaicism

Bahareh Ahmadi^1^, Tamas Revay^1^, Ruby Yoana Murcia-Robayo^2^, Ignacio Raggio^2^, Daniel A.F. Villagomez^1,3^, Mouhamadou Diaw^2^, Keith Colquhoun^4^ , W. Allan King^1,5^

**1** *Department of Biomedical Sciences, Ontario Veterinary College, University of Guelph, 50 Stone Rd E, Guelph, Ontario, Canada, N1G 4S7* **2** *Department of Clinical Sciences, Faculty of Veterinary Medicine, University of Montréal, 3200 Rue Sicotte, Saint-Hyacinthe, Quebec, Canada, J2S 2M2* **3** *Departamento de Producción Animal, Universidad de Guadalajara, Camino Ramón Padilla Sánchez 2100, Zapopan, Jal. 44600, Mexico* **4** *Colquhoun Equine Veterinary Services, 5151 Wellington Road 29, Rockwood, ON, Canada, N0B 2K0* **5** *Karyotekk Inc, Box636 OVC, 50 Stone Rd E, Guelph, Ontario, Canada, N1G 4S7*

Corresponding author: *Daniel A.F. Villagomez* (*dvillago@uoguelph.ca*)

Monosomy of the X chromosome, the most frequent sex chromosome abnormality associated with infertility in mares, has been investigated since early 1970’s. Affected mares may show one or more of the clinical symptoms such as short stature, undeveloped reproductive organs, and absent or irregular estrous cycle. It has been shown that some clinical features including fertility are correlated with the percentage of normal cells present. In our study, the presence of chromosomal mosaicism (XO/XX) in two mares (Irish Cob maiden, Standardbred) with fertility problem was determined using three independent techniques. Case No. 1 was phenotypically normal with normal external genitalia appearance, normal ovaries, and two tubular structures compatible with cervices on the pelvic floor. Case No. 2 was normal size with flaccid uterus, very small ovaries, and atonic cervix of normal size. Using chromosome-counting technique, fluorescent staining of interstitial C-band of X chromosome, and fluorescence *in situ* hybridization revealed 14.6% and 95% of X monosomy for each case, respectively. As the percentage of X monosomy in the first mare is low and the ovaries are functional, the fertility problem could be related to a failure of maternal recognition of pregnancy in response to movements of the embryo being limited to one uterine horn. Therefore, there are some possibilities for the production of a foal in this case. The second horse with 95% of X monosomy and very small ovary is infertile. This report highlights the importance of molecular cytogenetic techniques in cases of equine infertility to decide whether to keep breeding from such individuals.

## P31

### Chicken tandem repeats Ggal10 and Ggal20 are specific to different microchromosomes

Valeriia A. Volodkina, Maria M. Kulak, Aleksei S. Komissarov, Svetlana A. Galkina, Elena R. Gaginskaya, Alsu F. Saifitdinova

*Saint Petersburg State University, Universitetskaya emb. 7/9, Saint Petersburg, 199034, Russia*

Corresponding author: *Valeriia Volodkina* (*leravolo94@gmail.com*)

Repeats are large and significant part of genome in many organisms and are proved to be functionally important. The chicken (*Gallusgallus* Linnaeus, 1758) genome is one of the smallest among vertebrates. Its haploid size has been estimated to be 1.25 pg (1.223 Gbp). The karyotype (2n=78) includes 10 pairs of macrochromosomes, 28 pairs of microchromosomes, and a pair of sex chromosomes ZZ or ZW. Although the content of repetitive DNA in avian genomes is considerably less than in other classes of vertebrates, interspersed and tandem repetitive sequences occupy 31-35 % of the chicken genome. Guizard et al. (2016) noticed the different distributions of repeats in avian chromosomes when compared to other vertebrate genomes investigated so far. Apart from the most abundant telomere repeat, in avian genomes (as exemplified primarily in the chicken genome studies) there are many different, small families of satellite DNAs, copious in small chromosomes, which might label each small chromosome with a “satellite DNA code”. Since modern assembling techniques are not able to cope with tandem repeats, the way to solve the problem is to investigate them purposefully.

We have done research on identification and assessment of tandem repeats represented in the chicken *Gallusgallus* sequence raw archive (NCBI) for female (SRR958465) and male (SRR958466). The Lyrebird software was created for this purpose (Komissarov et al. 2018).

Isolated from raw reads, the new tandem repeat Ggal10 consists of 10-mers GCCCCATAGA. In female chicken genome this repeat constitutes a part of more than 2 Mb while in male genome it is less than 0.2 Mb. Published assembly of chicken genome and database of W-specific repeats don’t have the sequence of Ggal10. FISH on mitotic chicken chromosomes with specific oligonucleotide probe revealed the only pair of microchromosomes bearing Ggal10 in male and female karyotypes. There was no FISH signal on W chromosome.

The polymorphic tandem repeat Ggal20 consists of 20-mer AAATCCATAGCCATCATTGT. Its AT content is about 65%. There is less than 0.2 Mb representing this repeat in female genome and approx. 0.3 Mb in male one. In the actual chicken genome assembly this repeat is located on Z chromosome, but there were no FISH signals on sex chromosomes. We have revealed dispersed signals corresponding to Ggal20 on a few chicken microchromosomes.

These data contribute to the complete deciphering of the chicken microchromosome content.

**Acknowledgements**

The work was financially and technically supported by “Chromas” Research Resource Center and Theodosius Dobzhansky Laboratory of Saint-Petersburg State University, RFBR grant # 16-04-01823.

## P32

### Early cytomolecular diagnostics of the Holstein-Friesian breed heifers from heterosexual multiple pregnancies

Anna Kozubska-Sobocińska, Grzegorz Smołucha, Barbara Danielak-Czech, Wojciech Witarski

*Department of Animal Molecular Biology, National Research Institute of Animal Production, Krakowska 1, 32-083 Balice n. Krakow, Poland*

Corresponding author: *Wojciech Witarski* (*wojciech.witarski@izoo.krakow.pl*)

Based on hitherto studies, 82 to 95% of heifers from heterosexual twin pregnancies are infertile freemartins defined by 60,XX/60,XY karyotype. The frequency of such, according to many authors is a function of the breed. This anomaly is a result of vascular anastomosis and exchange of hematopoietic tissue between dizygotic twins of different sex and leads to extensive pathological changes in the female reproductive system, i.e., masculinization of sexual organs. The masculinization level is independent of the content of a cell line 60, XY adopted from the male sibling. The presented research is an attempt to determine the frequency of freemartinism in the HF-cattle population which can be further used for recommendations regarding early cytogenetic diagnostics and selection of heifers from heterosexual multiple pregnancies. In this study, we used 26 HF heifers (2 to 3 weeks old) and analyzed the metaphase chromosomes (minimum 100 metaphase plates per animal). In 24 heifers, leukocyte chimerism determined by the 60,XX/60,XY karyotype was revealed. The participation of the male cell line 60,XY in individual animals was in the range of 6% to 95%, whereas the normal karyotype, i.e., 60,XX was found in two heifers. The quality of cytogenetic analysis was confirmed by the study employing 12 microsatellite sequences, including BM1818, BM1824, BM2113, ETH3, ETH10, ETH225, INRA10, INRA23, SPS115, TGLA53, TGLA126, TGLA227, and heterosome markers such as AMELX and AMELY and SRY. The obtained results show that freemartinism is coupled to 92% of HF heifers from heterosexual multiple pregnancies and only 8% of heifers without the karyotype change can be qualified for further breeding and reproduction.

**Acknowledgements**

The work was financially supported from The Ministry of Agriculture and Rural Development funds, project no. 03-17-23-09.

## P33

### 3D architecture of japanese quail DNA repeats in interphase nucleus

Anna M. Zlotina^1^, Antonina V. Maslova^1^, Nadezda V. Kosyakova^2^, Thomas Liehr^2^, Alla V. Krasikova^1^

**1** *Saint Petersburg State University, 7-9 Universitetskaya Emb., St Petersburg 199034, Russia* **2** *Jena University Hospital, Institute of Human Genetics, Am Klinikum 1, 07747 Jena, Germany*

Corresponding author: *Anna M. Zlotina* (*anna-zlotina@yandex.ru*)

Japanese quail (*Coturnixc.japonica* Temminck et Schlegel, 1849), a galliform domestic species closely related to chicken, has typical avian karyotype that includes several pairs of macrochromosomes and numerous tiny microchromosomes (2n=78). The quail chromosomes are known to possess multiple heterochromatic segments such as centromeric regions and enigmatic blocks constituting short arms of submetacentric microchromosomes. Using lampbrush chromosome mechanical microdissection we previously generated FISH-probes specific to centromeric repeats of macrochromosomes (1-5) and repetitive elements from short arms of microchromosomes. Their karyotype distribution and precise positions on giant lampbrush chromosomes were subsequently investigated. In the present study we got insights into three-dimensional organization of the isolated repeats in quail interphase nucleus from embryonic and adult tissues. In particular, we showed that prominent DAPI-positive chromocenters contain repressive H3K9Me3 and HP1β chromatin markers typical for constitutive heterochromatin and accumulate repetitive DNA-sequences from short arms of quail microchromosomes. In all types of cells, clusters of microchromosomal centromeric BglII-repeats rim the chromocenters. In contrast, the centromere repeats of the largest macrochromosomes (1 and 2) are predominantly located in perinuclear heterochromatin, while the rest macro-centromeres are observed as patches on the periphery of chromocenters. Some differences of spatial nucleus architecture between cultured embryonic fibroblasts and cells from adult tissues were noted. To conclude, well-known specific morphology of quail interphase nuclei as compared to chicken is due to clustering of large blocks of repeats from short arms of microchromosomes.

**Acknowledgements**

The research was supported by a grant of the President of the Russian Federation (MK-1630.2017.4). The work was partially performed using experimental equipment of the Research Resource Centers “Chromas” and “Molecular and Cell Technologies” of St. Petersburg State University.

